# Glucose-Derived Carbon Materials via Hydrothermal Carbonization and Pyrolysis for Electrochemical Applications

**DOI:** 10.1021/acsomega.6c05437

**Published:** 2026-09-08

**Authors:** Behnam Jabbari Kalkhoran, Alessandro Chesini, Maida A. Costa de Oliveira, Filippo Marchelli, Roberto Verucchi, Michele Orlandi, Luca Fiori

**Affiliations:** † C3A-Center Agriculture Food Environment, University of Trento, Trento 38010, Italy; ‡ Department of Physics, University of Trento, Trento 38123, Italy; § Department of Civil, Environmental and Mechanical Engineering, University of Trento, Trento 38123, Italy; ∥ CNR-IMEM, Trento Unit c/o FBK, Trento 38123, Italy; ¶ Department of Civil, Chemical and Environmental Engineering, University of Genova, Genova 16145, Italy; □ School of Chemistry, Trinity College Dublin, Dublin 2, Ireland

## Abstract

Carbon materials are widely used in electrochemical technologies due to their tunable structure and chemical versatility. Hydrothermal carbonization (HTC) of biomass offers a sustainable synthesis route, yet quantitative links between processing conditions and material properties remain unclear. In this study, glucose-derived hydrochars were produced by systematically varying HTC temperature (180–210 °C), residence time (0.5–3 h), and precursor concentration. Increasing reaction severity promoted carbon retention in the solid phase while reducing soluble intermediates. Spectroscopic analyses (FTIR, XPS, Raman) revealed progressive heteroatom removal and development of sp^2^ carbon domains. Subsequent pyrolysis further enhanced structural ordering and reduced oxygen functionalities without altering morphology. Electrochemical measurements showed stable double-layer capacitance for pristine hydrochars (66 μF cm^–2^), with moderate improvement after pyrolysis (79 μF cm^–2^). Oxygen evolution reaction kinetics remained typical of metal-free carbons. Overall, this work establishes clear correlations among HTC conditions, carbon partitioning, structural evolution, and electrochemical behavior, supporting rational design of biomass-derived carbon materials.

## Graphical abstract

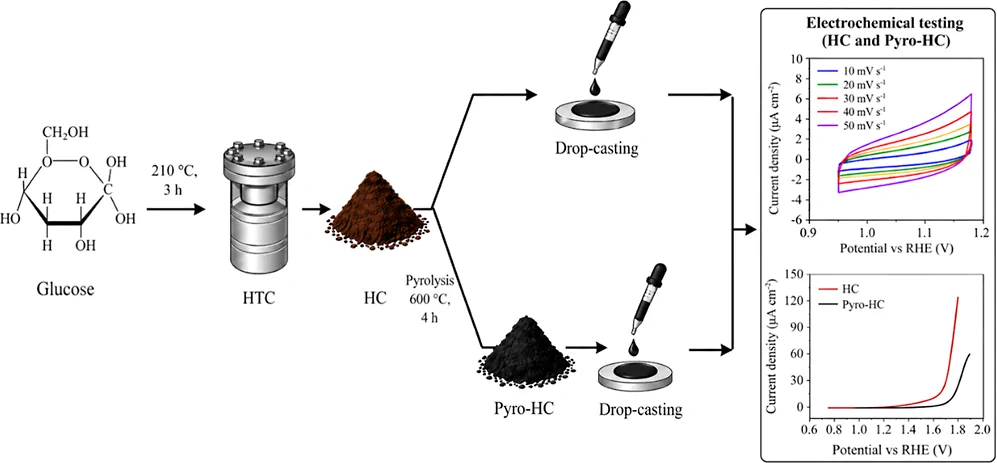

## 1 Introduction

Biomass is increasingly recognized as a sustainable, abundant, and renewable feedstock for producing value-added carbon materials, offering viable alternatives to fossil-derived carbons while mitigating environmental impacts associated with conventional carbon production.^1–3^ Among biomass-derived precursors, glucose is widely used as a model carbohydrate due to its high solubility, well-defined reaction chemistry, and suitability for systematic investigation of carbonization mechanisms.^4,5^ The use of glucose enables controlled studies of reaction pathways and provides a platform for designing carbon materials with predictable physicochemical properties.^6,7^ Hydrothermal carbonization (HTC) is a versatile thermochemical process that converts biomass into a carbon-rich solid (hydrochar) under moderate temperatures (typically 180–250 °C) and autogenous pressure.^5,7,8^ HTC mimics natural coalification processes on accelerated time scales, producing solid hydrochar, liquid organic intermediates, and gaseous byproducts such as CO_2_.^8–10^ The chemical transformations occurring during HTC are governed by dehydration, decarboxylation, polymerization, and aromatization reactions, which collectively drive carbon densification and heteroatom removal.^7,11,12^ The values of the operating conditions (temperature, residence time, and biomass-to-water ratio) together define the hydrothermal reaction severity.^13,14^ Despite extensive research on hydrochar synthesis and formation, reproducible control over carbon partitioning, structural evolution, and interfacial electrochemical behavior remains challenging. In particular, a systematic understanding of how combined HTC parameters govern carbonization pathways and surface chemistry evolution, and how these changes influence electrochemical response, remains limited. Most previous studies have focused primarily on yield optimization or morphological description rather than establishing quantitatively the effect of structure–property and electrochemistry relationships.^9,15,16^ Recent studies have explored various biomass-derived carbon materials as potential electrodes for electrochemical energy storage and conversion, demonstrating their effectiveness in supercapacitors, batteries, and catalytic reactions.^17–23^ However, most of these investigations employ structurally complex biomass sources such as agricultural residues or lignocellulosic wastes,^17,18^ rather than well-defined molecular precursors like glucose, limiting the mechanistic understanding of carbonization pathways. Previous studies have focused primarily on hydrochar yield and morphology,^24–26^ while direct correlations among carbon structure, surface chemistry, and intrinsic electrochemical behavior remain poorly understood.^22,27,28^ The use of glucose as a chemically well-defined precursor provides a controlled platform for establishing these structure and electrochemistry relationships, which are difficult to isolate in complex structures of biomass feedstocks. Hydrochars possess intrinsic characteristics that make them attractive for fundamental electrochemical studies, including tunable surface chemistry, defect-rich carbon structures, and electrochemically accessible surfaces. Importantly, intrinsic electrochemical behavior can be influenced by structure and heterogeneous surface functionalities, and controlling these properties becomes essential to investigate and translate electrode/electrolyte redox processes. Post thermal treatment can further modify the hydrochar structure by promoting the removal of oxygen-containing functional groups and enhancing sp^2^ carbon domain formation, thereby influencing interfacial charge storage and electron transport characteristics,^3,29^ typical of materials for electrochemical applications.

The oxygen evolution reaction (OER) remains one of the major limitations for hydrogen production in electrolyzers, due to its sluggish kinetics, high overpotential,^30,31^ and sensitivity to the surface chemistry and pH dependence.^32,33^ In this study, we systematically investigate the combined effects of HTC temperature, residence time, and biomass-to-water ratio on the structural and chemical evolution of glucose-derived hydrochars. Carbon partitioning among solid, liquid, and gaseous phases was quantified to evaluate the carbonization efficiency. Structural ordering, surface chemistry, and defect evolution were examined by using complementary spectroscopic techniques. The electrochemical behavior of hydrochar and pyrolyzed hydrochar was evaluated in different electrolyte pHs to elucidate interfacial charge transfer, conversion, and storage. OER is our probe reaction to investigate how hydrothermal carbonization and pyrolysis treatment influence the intrinsic electrochemical activity of glucose-derived hydrochars rather than as a model of electrocatalytic performance. Therefore, we define a reproducible hydrothermal carbonization-pyrolysis framework for controlling hydrochar surface chemistry and structure, to establish mechanistic links among the severity of the hydrothermal reaction of glucose, the development of the carbon structure, the evolution of surface chemistry, and the electrochemical response of the oxygen evolution reaction. The findings provide fundamental insight into biomass-derived carbon formation and demonstrate that controlled chemical densification, rather than empirical performance optimization, can be used to design functional carbon materials for electrochemical application, highlighting a viable pathway for biomass valorization into high-value carbon materials for applications in energy conversion and storage.

## 2 Materials and Methods

The feedstock chosen for this study was dry glucose (C_6_H_12_O_6_–99%) which, as the chemicals NaCl (99%), NaOH (98%), H_2_SO_4_ (98%), and acetone (HPLC grade ≥99.9%), was supplied by Sigma-Aldrich. Alumina slurries (1, 0.3, and 0.05 μm particle size) and polishing cloths (MicroCloth and Nylon Polishing cloths) were purchased from Ted Pella and Buehler, respectively. Glassy carbon plates (GC) (Sigradur, 2 × 1 cm^2^) were purchased from Hochtemperatur-Werkstoffe GmbH. Aquivion 25% in water (PFSA eq wt. 980 g/mol SO_3_H) was obtained from Sigma-Aldrich.

### 2.1 Materials Preparation

#### 2.1.1 Preparation of Hydrochar

Hydrochar materials were synthesized in a 50 mL stainless steel reactor following the experimental procedure previously reported.^34^ Glucose and distilled water were fed to the reactor with mass ratios (here referred to as biomass to water ratio–B/W–to follow typical scientific literature standards) of 0.25, 0.5, and 1 while maintaining the reactor filling level below 70% of its total capacity. Prior to heating, the reactor was purged with nitrogen (N_2_) to remove oxygen and ensure an inert atmosphere. The sealed reactor was heated using an electric resistance heating system equipped with a temperature controller. Reaction time was recorded after the reactor reached the target temperature (180 or 210 °C), with residence times of 0.5 and 3 h. During the reaction, self-generated pressure developed in accordance with the vapor pressure of water. After the designated residence time elapsed, the reactor was rapidly cooled by immersion in a cold-water bath until the internal temperature dropped below 55 °C. Different combinations of B/W, temperature, and reaction time were investigated. Samples were denoted as xC-yh-B/W = z, where x is the temperature (°C), y is the reaction time (h), and z is the glucose-to-water ratio. All experimental conditions investigated are summarized in Table S1. All hydrothermal carbonization experiments were conducted in triplicate, and results are presented as mean ± standard deviation.

#### 2.1.2 Product Separation

Gaseous products were released by carefully opening the HTC reactor outlet valve after the reactor was cooled. The gas volume was measured by water displacement using a graduated cylinder filled with water and closed at the top; the difference between the initial and final water levels was recorded. In order to calculate the gas yield and carbon balance, the ideal gas law was used and the gas phase was assumed to consist entirely of CO_2_. This is a simplifying assumption commonly adopted in the literature, which is based on experimental data testifying to an HTC gas composition largely dominated by CO_2_ (>96%).^35,36^

The resulting HTC slurry, containing hydrochar and HTC liquor, was then removed from the reactor. Hydrochar was separated from the liquor by vacuum filtration using a 0.45 μm quantitative filter membrane. The solid residue collected on both the filter membrane and reactor walls was transferred to an oven and dried at 105 °C for 24 h to remove residual moisture. The liquid filtrate (HTC liquor) was stored at 4 °C for subsequent analyses. A schematic of the HTC process and yield calculations is presented in Figure S1 and equations (eq S1–S3): SY, GY, and LY stand for solid, gas, and liquid yields, respectively.

#### 2.1.3 Thermal Treatment of Hydrochar

Based on the SY value and its chemical features (Sections 3.1 and 3.2), the hydrochar produced at 210 °C for 3 h with a B/W ratio of 0.5 was selected for post-treatment by pyrolysis. The selected hydrochar (HC) was placed in a quartz crucible and thermally treated at 600 °C for 4 h under nitrogen in a Carbolite Gero TF1–1200 furnace (heating rate to reach the set point temperature equal to 7 °C min^–1^). Heating and cooling were performed under continuous inert gas flow to minimize oxidation. The schematic of the thermal program is shown in Figure S2. The resulting material is referred to as pyrolyzed hydrochar (Pyro-HC).

### 2.2 Analytical, Surface Chemistry, and Morphology Characterizations

#### 2.2.1 Analytical Characterization

Elemental composition, through quantification of carbon and hydrogen in the solid samples, was measured using an LECO 628 Elemental Analyzer, in accordance with ASTM D5375. Since the glucose used contained neither nitrogen nor ash, the oxygen content was ascertained by utilizing the difference (O % = 100–C %–H %). Alongside the elemental analysis test, the total organic carbon (TOC) test was performed by SKALAR 2CA17910 in accordance with the ASTM D7573 standard to measure the carbon content in the liquor filtered after HTC.

#### 2.2.2 Surface Chemistry and Morphology Characterizations

Scanning electron microscopy (SEM) images of HC samples were collected using a Zeiss Supra 40 field emission scanning electron microscope (Carl Zeiss SMT GmbH) operating in vacuum at 10^–6^ Torr, equipped with a secondary electron detector and working at an acceleration voltage of 2.5 kV. Fourier transform infrared spectroscopy (FTIR) was performed using an Agilent Cary 630 FTIR spectrometer. The acquisition range was between 4000 and 400 cm^–1^. Raman spectroscopy analysis was performed using a LabRAM Aramis Jobin-Yvon Horiba confocal μ-Raman system equipped with a 633 nm laser source limited to a maximum of 10% power to avoid sample damage. Acquisitions were performed with 1800 lines/mm grating. The signal was acquired with a CCD in the range of 1250–1700 cm^–1^.

X-ray photoelectron spectroscopy (XPS) was carried out in an ultrahigh-vacuum (UHV) chamber, equipped with a non-monochromatized X-ray source (KαMg = 1253.6 eV) and a VSW HA100 hemispherical analyzer (with PSP electronic power supply and control), leading to a total energy resolution of 0.86 eV. The binding energy (BE) scale of XPS spectra was calibrated by using the Au 4f peak at 84.0 eV as a reference. Core level analysis has been performed by Voigt line-shape deconvolution after background subtraction of a Shirley function. It is worth mentioning that XPS cannot reveal the presence of hydrogen. BEs for HC were shifted by −1.6 eV due to charging during XPS analysis.

### 2.3 Electrochemical Characterizations

#### 2.3.1 Fabrication of Hydrochar-Based Electrodes

A 0.7 cm^2^ glassy carbon (GC) electrode was used as the substrate for hydrochar materials. Prior to use, the GC surface was sequentially polished with alumina slurries of 1.0, 0.3, and 0.05 μm particle sizes on appropriate polishing cloths until a mirror-like finish was obtained, following previously reported procedures.^37^ Between each polishing step, the electrodes were cleaned in deionized water using an ultrasonic bath for 10 min to prevent cross-contamination and were then dried at room temperature. Before each drop-casting modification, the GC disks were sonicated in isopropanol and dried. The deposition ink was prepared by dispersing 4 mg of hydrochar in 310 μL of acetone, followed by sonication for 20 min. After that, 100 μL of deionized water (R = 18 M Ω/cm) was added, and the dispersion was sonicated for another 20 min. Finally, 50 μL of Aquivion were added to enhance electrode stability and powder adhesion to the substrate, and the resulting ink was sonicated for an additional 10 min. The electrode was fabricated by drop-casting 20 μL of this dispersion of HC or Pyro-HC onto the GC substrate, yielding a loading of 0.25 mg cm^–2^ (see Figure S3).

#### 2.3.2 Electrochemical Characterizations

The electrochemical experiments were conducted in a three-electrode-cell configuration. Prior to the tests, a quartz tube was filled with 100 mL of an electrolytic solution and consecutive addition of Ag/AgCl (3 M KCl) as the reference electrode (RE), a graphite rod as the counter electrode (CE), and glassy carbon (GC) coated with HC and Pyro-HC films as the working electrode (WE). The measurements were carried out through a Gamry 1000 Interface potentiostat, and all the potential values were shifted to the reversible hydrogen electrode (RHE) by using the Nernst equation: V_RHE_ = V_Ag/AgCl_ + 0.197 + 0.0592pH, where V_Ag/AgCl_ represents the experimental potential value read by the potentiostat and +0.197 is the standard potential of the Ag/AgCl reference against the standard hydrogen electrode (SHE). The electrochemical performance of the bulk electrodes was evaluated in basic, neutral, and acidic environments. The alkaline electrolyte solution was made of 0.1 M NaOH (pH = 12.4), the neutral one was 0.1 M NaCl (pH = 5.4), and the acidic one was 0.1 M H_2_SO_4_ (pH = 1.5). Preceding each measurement, N_2_ gas was purged inside the electrolyte to remove oxygen and other interfering gases.

## 3 Results and Discussion

### 3.1 Hydrochar Production

HTC of glucose was investigated over a range of temperatures (180 and 210 °C), biomass-to-water ratios (B/W = 0.25, 0.5, and 1), and residence times (0.5 and 3 h). Since the solid fraction was used for the subsequent structural, chemical, and electrochemical analyses, solid yield (SY) is reported as the main yield parameter in Figure 1. The corresponding measured gaseous yield (GY) and calculated liquid yield (LY), obtained by difference according to eq S3, are reported in Table S2.

**Figure 1:**
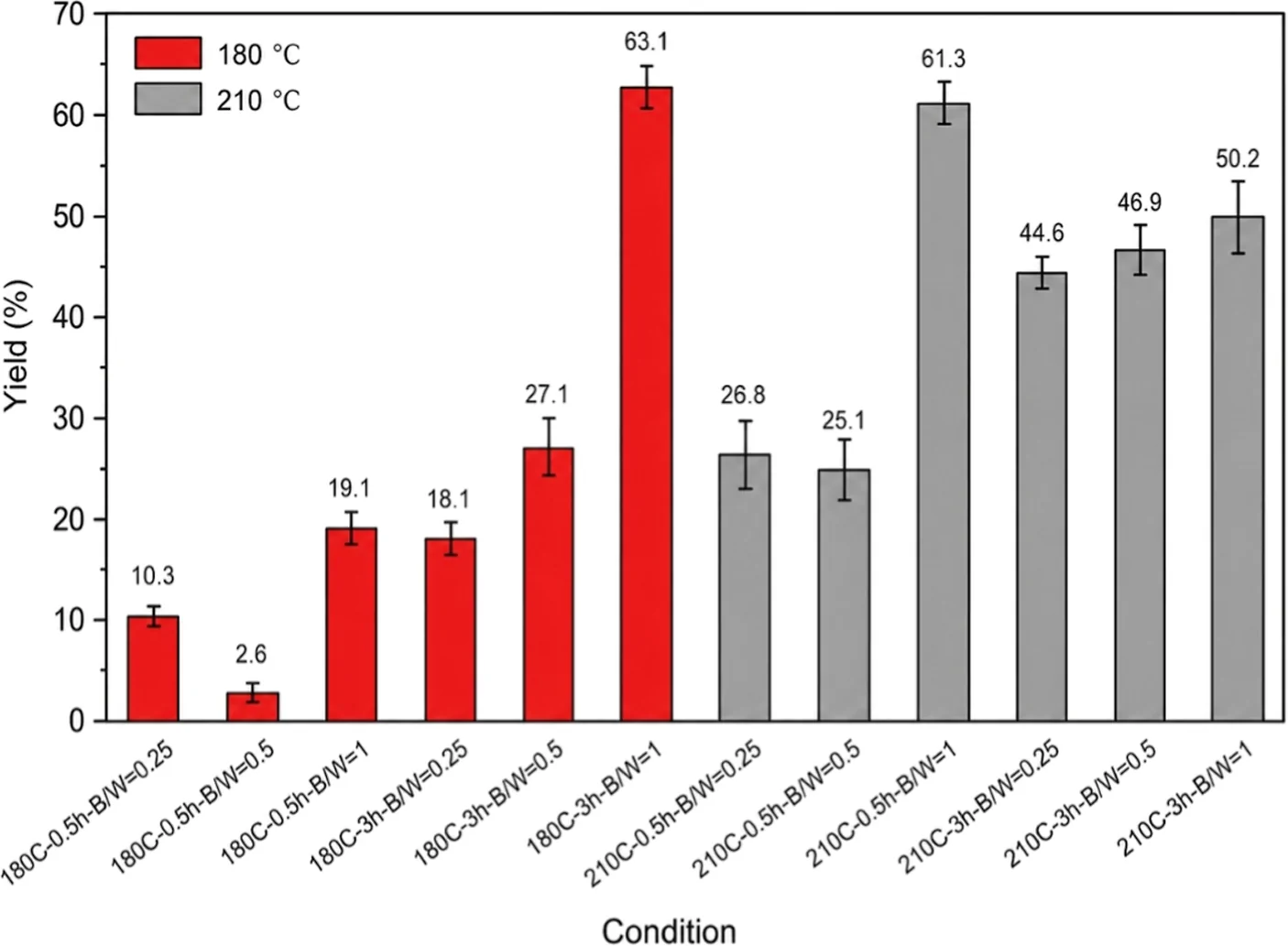
Effect of HTC temperature, residence time, and biomass-to-water ratio (B/W) on solid yield (SY) during hydrothermal carbonization of glucose.

At 180 °C and short residence times (0.5 h), SY was low across all B/W ratios, peaking at 19.1% for B/W = 1. At residence time of 3 h at the same temperature, SY significantly improved, reaching 63.1% for B/W = 1. At 210 °C, the solid yield resulted in a notable increase with respect to 180 °C for all the tested conditions but one (3 h and B/W = 1): notably at 210 °C and B/W = 1, SY was 61.3% after 0.5 h and 50.2% after 3 h. These results indicate that increasing reaction temperature and duration promotes hydrothermal carbonization reactions, leading to higher solid-phase carbon retention and enhanced carbon densification.

The B/W ratio appears to influence hydrochar formation mainly through the concentration of glucose-derived intermediates available for the condensation and polymerization reactions. However, in the present data set, its effect was coupled with temperature and residence time, which remained the main drivers of oxygen removal and structural condensation.^6,24,38^

### 3.2 Elemental Analysis

The elemental composition of the hydrochar samples was determined under varying reaction conditions (the temperature, B/W ratio, and residence time). The results are summarized in Figure 2.

**Figure 2:**
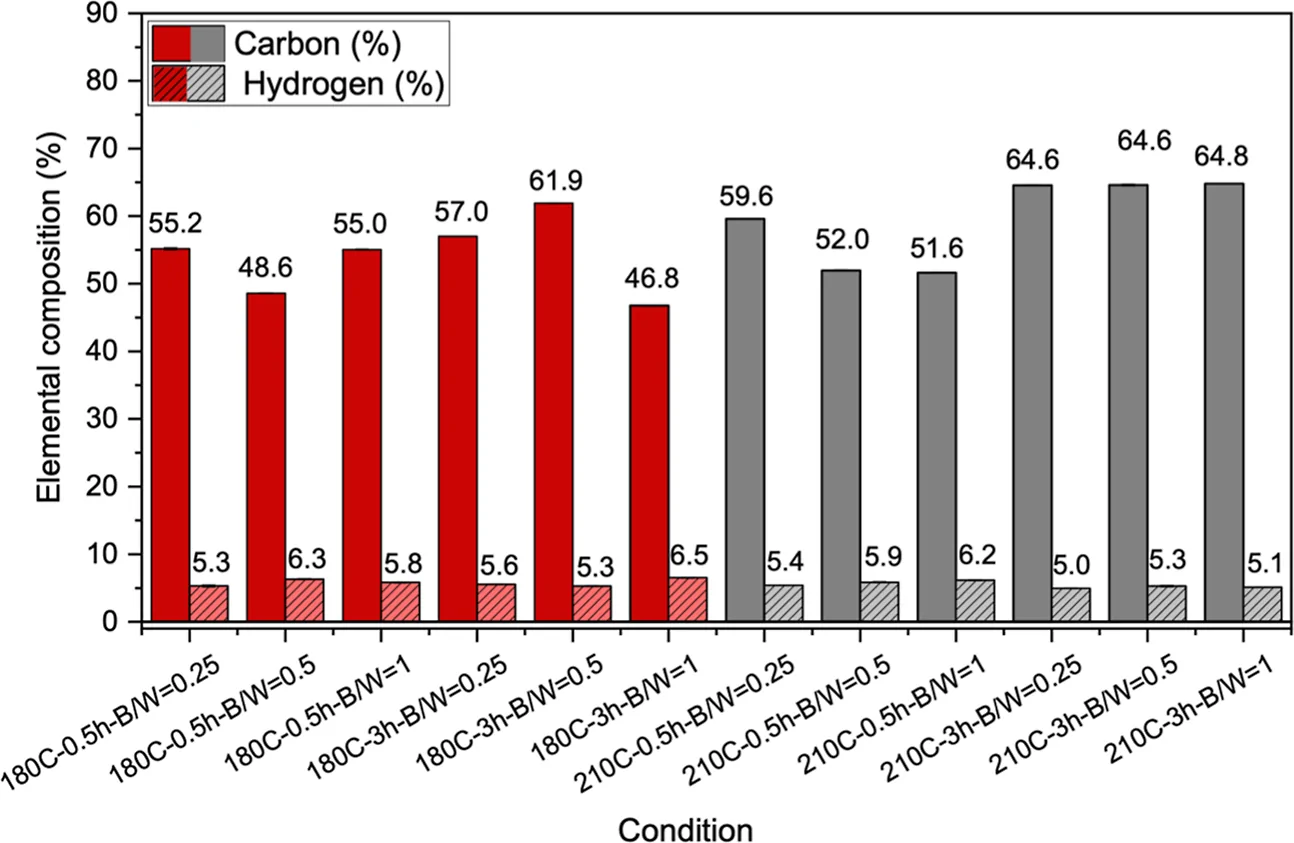
Elemental composition of hydrochar samples under various reaction conditions, analysis repeated at least 2 times with ±1% error.

Carbon content ranged from 46.8% to 64.8%, with the highest value observed for the sample prepared at 210 °C, 3 h, and B/W = 1. Increasing the reaction temperature from 180 to 210 °C led to a slight increase in carbon content, particularly at longer residence times, indicating that higher temperature promotes dehydration, decarboxylation, and aromatization reactions, driving progressive oxygen removal and structural carbon densification.^7,12^ This trend is consistent with the expected loss of oxygen and hydrogen functional groups as volatile species under hydrothermal conditions.^9^ A slight decrease in hydrogen content was relatively uniform across all samples, from 5.0% to 6.5%, and was observed with increasing temperature and residence time, resulted from the release of hydrogen compounds such as water and volatile organics, due to dehydration at 210 °C. Nitrogen content was minimal, not exceeding 0.2% in any sample, consistent with the nitrogen-free nature of glucose and the inert environment maintained by the nitrogen atmosphere. Thus, trace nitrogen possibly originates from minor feedstock impurities or elemental analyzer detection limits.

Carbon partitioning between solid and liquid phases was evaluated by using TOC analysis and atomic ratio mapping. TOC quantified dissolved organic carbon remaining in the aqueous phase (HTC liquor) after hydrothermal carbonization and filtration, which primarily consists of soluble intermediates such as furfurals, hydroxymethylfurfural (HMF), phenolic species, and low-molecular-weight organic acids.^7,24^ As such, the TOC serves as an indirect indicator of carbon retention and reaction progression during hydrothermal carbonization. Low TOC values indicate that carbonaceous intermediates have predominantly undergone dehydration, condensation, and aromatization reactions, thereby favoring carbon retention in the hydrochars. In contrast, elevated TOC concentrations indicate enhanced formation and stabilization of soluble oxygenated fragments resulting from partial degradation or incomplete secondary polymerization of glucose-derived intermediates.^39^

As demonstrated in Table 1, TOC values varied substantially depending on temperature, residence time, and B/W ratio. Samples treated for 3 h consistently exhibited lower TOC concentrations compared to those processed for 0.5 h, indicating that extended residence time promotes secondary polymerization and structural stabilization of the solid carbon matrix. The increased temperature from 180 to 210 °C also resulted in a pronounced reduction in TOC, particularly at 3 h, where values decreased to 21.2 and 29.0 g L^–1^ for B/W ratios of 0.25 and 0.5, respectively. To further elucidate the structural evolution of the hydrochars, Van Krevelen analysis was performed using the atomic H/C and O/C ratios calculated from the elemental composition data. The equations used for atomic ratio determination and the corresponding Van Krevelen diagram are provided in the Supporting Information (Equations S4 and S5 and Figure S4).

**Table 1:** TOC Values of HTC Liquor Samples after Filtration and the Atomic Ratios Obtained from the Elemental Composition of Hydrochars

synthesis condition	TOC (g/L)	atomic ratio	
		H/C	O/C
180 °C–0.5 h–B/W = 0.25	111.8	1.15	0.54
180 °C–0.5 h–B/W = 0.5	152.6	1.54	0.70
180 °C–0.5 h–B/W = 1	230.7	1.26	0.53
180 °C–3 h–B/W = 0.25	85.9	1.17	0.49
180 °C–3 h–B/W = 0.5	127.6	1.02	0.40
180 °C–3 h–B/W = 1	71.32	1.65	0.75
210 °C–0.5 h–B/W = 0.25	76.4	1.08	0.44
210 °C–0.5 h–B/W = 0.5	112.0	1.35	0.61
210 °C–0.5 h–B/W = 1	231.4	1.43	0.61
210 °C–3 h–B/W = 0.25	21.2	0.92	0.35
210 °C–3 h–B/W = 0.5	29.0	0.98	0.35
210 °C–3 h–B/W = 1	not available^a^	0.94	0.35

a The test had no recoverable liquor.

The calculated H/C and O/C values for each sample are included in Table 1 alongside the TOC results to facilitate a correlation between carbon partitioning and structural transformation. The Van Krevelen diagram reveals a systematic downward and leftward shift with higher temperature and residence time, corresponding to lower H/C and O/C ratios, indicating that the temperature is the most significant factor forming hydrochar. The products are changed in the direction of coal, particularly the samples produced at 210 °C for 3 h at different B/W, that exhibit the lowest O/C (0.35) and H/C (0.94–0.98) ratios, due to enhanced dehydration, decarboxylation, polymerization, and aromatization reactions.^40^ This progressive reduction in oxygen content is evidence of higher carbon densification and structural condensation under more severe HTC conditions.^9,24^ Particularly, the lowest O/C ratios coincide with the lowest TOC values, confirming that enhanced carbon incorporation into the solid phase occurs simultaneously with reduced accumulation of soluble intermediates in the liquid phase.^9,24,41^

The condition 210 °C–3 h–B/W = 1 requires a separate note. After reaction and separation, no free recoverable liquor was available for TOC analysis. This does not imply that the calculated LY was zero because LY is calculated by difference from SY and GY and represents the amount of glucose derivatives solubilized in the liquid phase. Rather, under this condition, the high precursor concentration and severe treatment produced a solid-rich residue in which the liquid fraction was completely retained and not filtered out during filtration. For this reason, TOC is not reported for this sample in Table 1.

### 3.3 Surface Chemistry

The hydrochar composition was investigated using a combination of spectroscopic methods. FTIR spectra of hydrochar reveals progressive structural condensation and chemical transformation with increasing temperature and residence time (see Figure S5). The broad O–H stretching band (3600–3200 cm^–1^) decreases in intensity, indicating dehydration and condensation reactions, in agreement with the decreasing O/C ratios observed in the Van Krevelen diagram.^6,24^ Aliphatic C–H bands (3000–2800 cm^–1^) also diminish with the degradation of hydrogen-rich aliphatic structures,^9,10^ which correlates with the reduction in H/C ratios. The aromatic C=C stretching bands (1600–1400 cm^–1^) intensify, indicating aromatization via cyclization and polycondensation reactions.^6^ Carbonyl (C=O, 1750–1650 cm^–1^) and ether/alcohol (C–O, 1200–1000 cm^–1^) bands provide additional insight into oxygen-containing functionalities and residual etheric structures, which partially persist across the hydrochars.^3,9^ The bands at 800–700 cm^–1^ are attributed to aromatic C–H out-of-plane bending vibrations^9^ and evidence for the presence of furanic fragment^42^ and for the presence of phenolic fragments in the hydrochar.^6^ The peaks and their corresponding assignments are shown in Figure S5 and Table S3.^9^ Therefore, the results suggest that temperature and residence time promote oxygen elimination, structural condensation, and aromatization, producing more carbon-dense hydrochars. Based on these characteristics, the hydrochar produced at 210 °C for 3 h (B/W = 0.5), which exhibited low O/C and H/C ratios along with the HTC liquor TOC value, was selected for post-thermal treatment.

The effect of post-thermal treatment on the selected hydrochar was again evaluated by FTIR spectroscopy, as shown in Figure 3. Compared with the untreated hydrochar, the pyrolyzed sample exhibits further attenuation of the O–H stretching band, indicating enhanced dehydration and condensation. Aliphatic C–H bands decrease further, consistent with additional dehydrogenation and removal of hydrogen-rich moieties.^3^ Aromatic C=C stretching bands intensify after pyrolysis, confirming continued aromatization and growth of conjugated domains. Carbonyl decreases relative to the hydrochar, due to decarboxylation, while C–O stretching bands remain detectable.^43,44^ Thus, enhanced carbon retention and a higher degree of structural condensation make Pyro-HC a suitable candidate for subsequent electrochemical evaluation.

**Figure 3:**
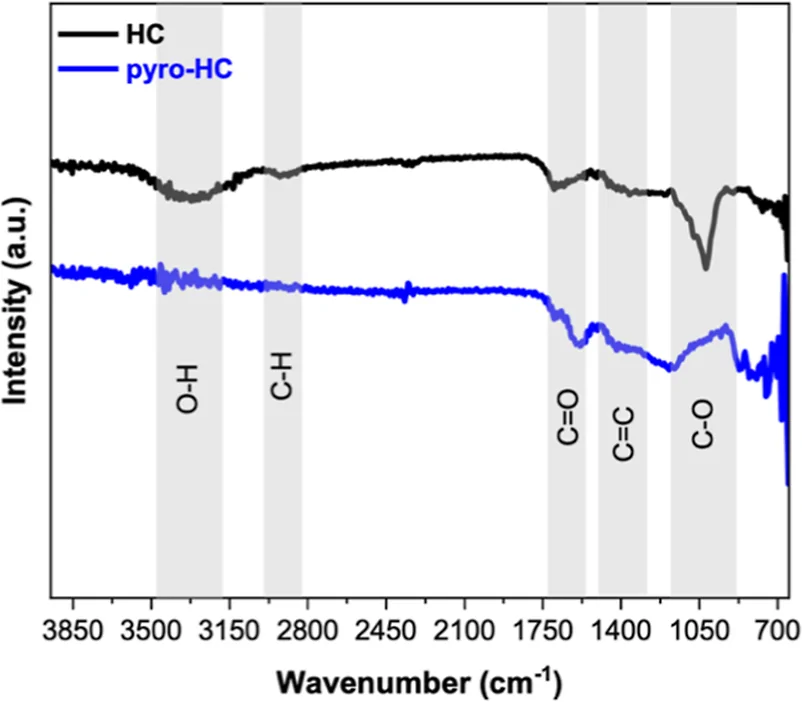
FTIR spectra of sample HC (black) and Pyro-HC (blue).

Changes in the surface chemistry were also proven via XPS. High-resolution C 1s and O 1s spectra of the as-prepared HC and Pyro-HC, along with the deconvolution results, are summarized in Table 2 and shown in Figure 4.

**Table 2:** Quantitative Data from XPS Analysis of Both Samples: The Percentage of Each Component Refers to the Single Total Core Level Area^a^

		HC		Pyro-HC	
core level	component	BE (eV)	% on core level area	BE (eV)	% on core level area
**C 1s**	sp^2^ graphite			284.42	82.00
	sp^3^	285.00	60.32		
	C–O	286.10	20.86	286.29	6.56
	C=O	287.02	12.54		
	plasmon			290.27	2.96
	C-OOR	288.48	6.28	288.40	8.48
**O 1s**	C-OOR	531.22	6.82		
	C=O	532.17	38.94	532.03	34.46
	C–O	533.49	54.24	533.44	65.54
**C/O ratio**			**5.04**		**22.92**

a The atomic C/O ratio is also shown.

**Figure 4:**
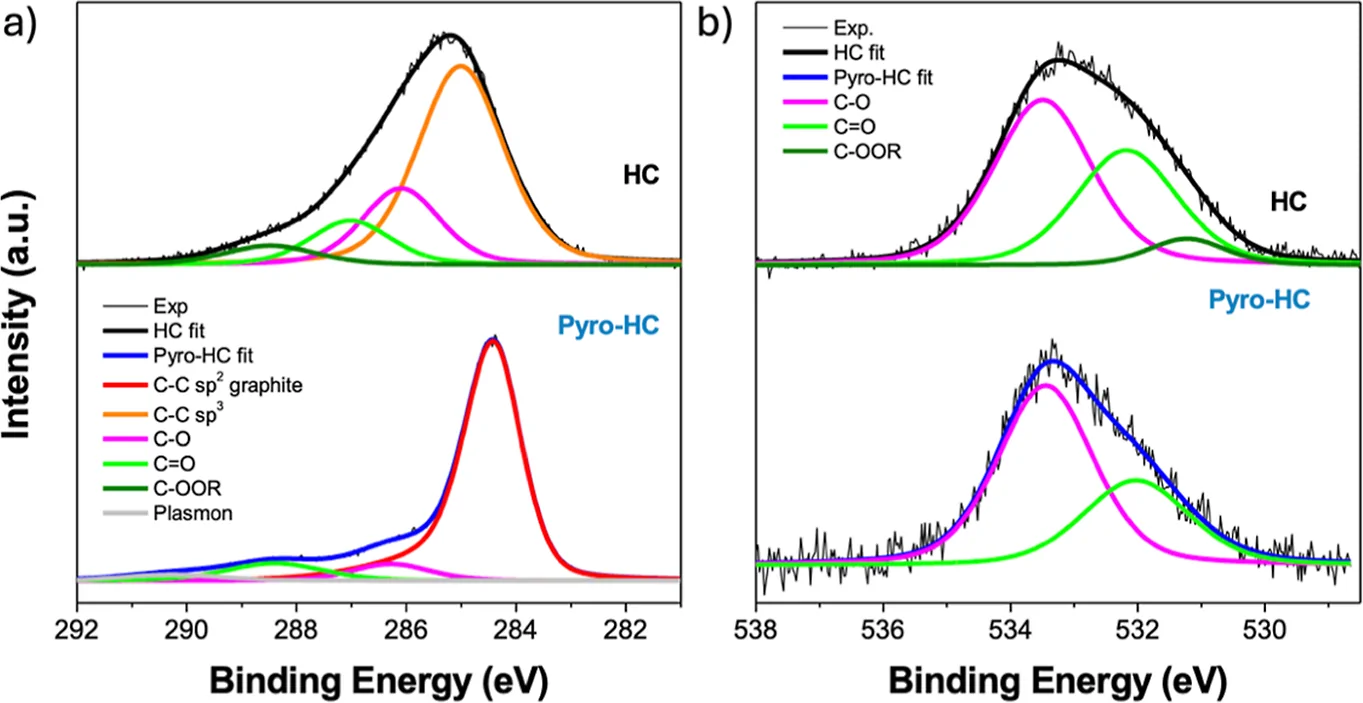
C 1s (a) and O 1s (b) core level analysis of HC and Pyro-HC (top and bottom curves, respectively). The spectra are background subtracted and normalized in height.

XPS analysis revealed the presence of carbon and oxygen, leading to C/O ratios for HC and Pyro-HC of 5.0 and 22.9, confirming the effective removal of surface oxygen during pyrolysis.^45,46^ The deconvolution of C 1s spectra for HC shows a mixture of sp^3^ carbon and oxygenated species, including C–O, C=O, and C–OOR functionalities.^47–49^

The concentration of C–O and C=O groups resulted from prolonged HTC 210 °C, conditions beneficial for the aldol condensation and etherification reactions of furan rings.^24^ In contrast, the C 1s core level in Pyro-HC exhibits a dominant sp^2^ carbon component at 284.4 eV, characteristic of graphitic domains, with no detectable sp^3^ carbon and carbonyl groups. This is consistent with atomic ratios from elemental composition, in which the O/C ratio gradually decreased because of the dehydration of hydrochar.^9,24,50,51^ The O 1s core level for HC shows the presence of three peaks, related to aliphatic carbon (C–O), carboxyl bonds (O=C), and oxygen single-bonded to aromatic carbon (C–OOR), in agreement with previous C 1s results.^3,6,51^ O 1s analysis indicates that HC possesses a significant oxygen content associated with C–O and C–OOR groups (about 60%). The O 1s core level for Pyro-HC is composed of only C–O and C=O groups, once more in agreement with C 1s characteristics. These XPS results align closely with the FTIR observations in Figure 3.

Raman spectroscopy was employed to further assess the structural organization of HC and Pyro-HC. Both samples exhibit characteristic D (ca. 1350 cm^–1^) and G (ca. 1580 cm^–1^) bands, corresponding to disordered carbon and graphitic sp^2^ domains, respectively, as shown in Figure 5.^52,53^ The HC shows relatively broad and weak D and G signals superimposed on a strong background, indicating a partially amorphous structure with a density of defects and residual oxygen-containing moieties. This behavior is present for most hydrochars with lower O/C and H/C ratios, while Pyro-HC exhibits more intense and well-defined D and G bands in Figure 5, along with a significant reduction of the background signal, as demonstrated in Figure S7. The Raman D band originates from the breathing modes of sp^2^ carbon atoms in both aromatic and olefinic molecules. It is assigned to ring breathing vibrations in benzene or condensed benzene rings in amorphous or partially hydrogenated carbons,^9,52^ which is consistent with hydrochar aromatization observed in FTIR results. In contrast, the G band corresponds to the in-plane stretching vibration of sp^2^-hybridized C=C bonds and is characteristic of graphitic carbon, arising from both aromatic domains and conjugated sp^2^ and olefinic molecules.^9^

**Figure 5:**
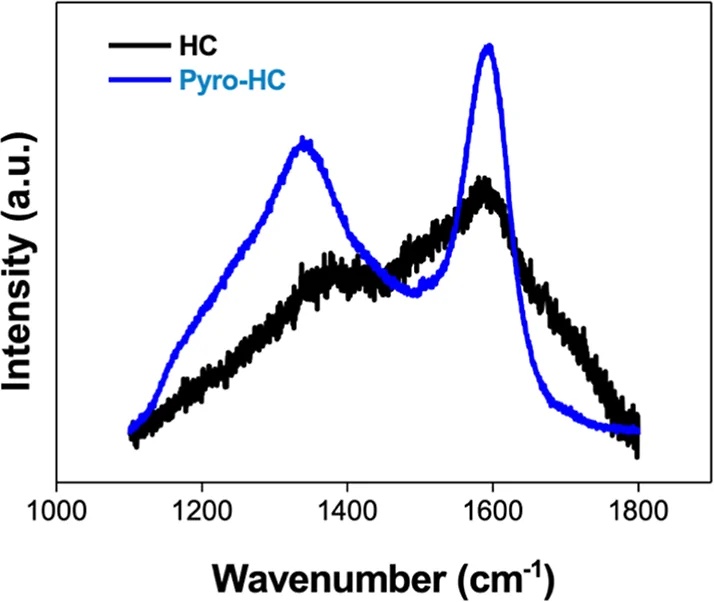
Raman spectroscopy of HC (black line) and Pyro-HC (blue line).

The intensity ratio between the D and G bands (I_D_/I_G_) is widely employed as a structural parameter to evaluate the ordering of carbon materials. The narrow and sharped G band, clearly visible after pyrolysis, is attributed to in-plane C=C sp^2^ stretch vibrations, which may arise in aromatic hydrocarbons.^3^ Alongside, the D band position shifts to lower wavenumbers after thermal treatment, from 1368 to 1357 cm^–1^, indicating progressive structural order and growth of sp^2^ carbon domains, consistent with the model proposed previously.^52^ Figure S6 shows that at 180 °C, only samples with a residence time of 3 h and a B/W ratio of 1 exhibit clearly resolved D and G bands. At 210 °C with a residence time of 3 h, both Raman bands are detected irrespective of the B/W ratio, suggesting enhanced structural order at higher treatment temperatures. Increasing the B/W ratio induces a further D band downshift of ca. 10 cm^–1^, whereas the G band position remains nearly constant at approximately 1585 cm^–1^. The stability of the G band position, combined with D band evolution, indicates the development of nanocrystalline carbon domains. The measured I_D_/I_G_ ratios increase slightly from 0.68 to 0.72 after thermal treatment at 600 °C. According to the amorphization trajectory as previously reported,^9,52^ in the nanocrystalline carbon regime, the D band intensity is not governed by crystallite boundary length but instead scales with the area of sp^2^ aromatic clusters. Consequently, the I_D_/I_G_ ratio is proportional to the number of six-membered aromatic rings within nanoclusters, suggesting a slight enlargement of aromatic domains, consistent with gradual structural condensation rather than extensive graphitization. These results indicate structural reorganization of the carbon matrix and defect reduction. This interpretation is consistent with the FTIR results, which show attenuation of O–H, C–O, and C=O bands, with XPS analysis revealing a substantial decrease in oxygen content together with an increase in sp^2^ carbon contributions after pyrolysis.

### 3.4 Morphology

The influence of chemical evolution on physical morphology was evaluated using SEM analysis, with the images displayed in Figure 6. HC and Pyro-HC exhibit spherical morphologies with typical micro- and nanospheres, mostly lying in the 1–2 μm range, expected for materials from hydrothermal carbonization of carbohydrates.^3,6,24,54^ No significant morphological differences are observed between HC and Pyro-HC, indicating that the post-pyrolysis treatment at 600 °C does not alter the spherical architecture, with only minor variation in the microsphere size distribution, as shown in Figure S12. Most spheres are interconnected, forming channels and interspherical voids within the structure. However, these interconnections cannot be strictly classified as intrinsic pores, as the surface of the spheres remain relatively smooth up to thermal treatments of approximately 600 °C. Above this temperature, distinct surface features begin to develop, suggesting the onset of surface texturing and structural modification, such as features and voids, as previously reported.^3^ Future analysis like gas adsorption will complement intrinsic micro- or nanospheres and morphology understanding. The combined spectroscopic and compositional analyses indicate progressive carbon densification, oxygen elimination, and sp^2^/sp^3^ domain growth with increasing hydrothermal reaction severity. These transformations are expected to modulate interfacial charge transfer and electrolyte interaction behavior (see Figure 7).

**Figure 6:**
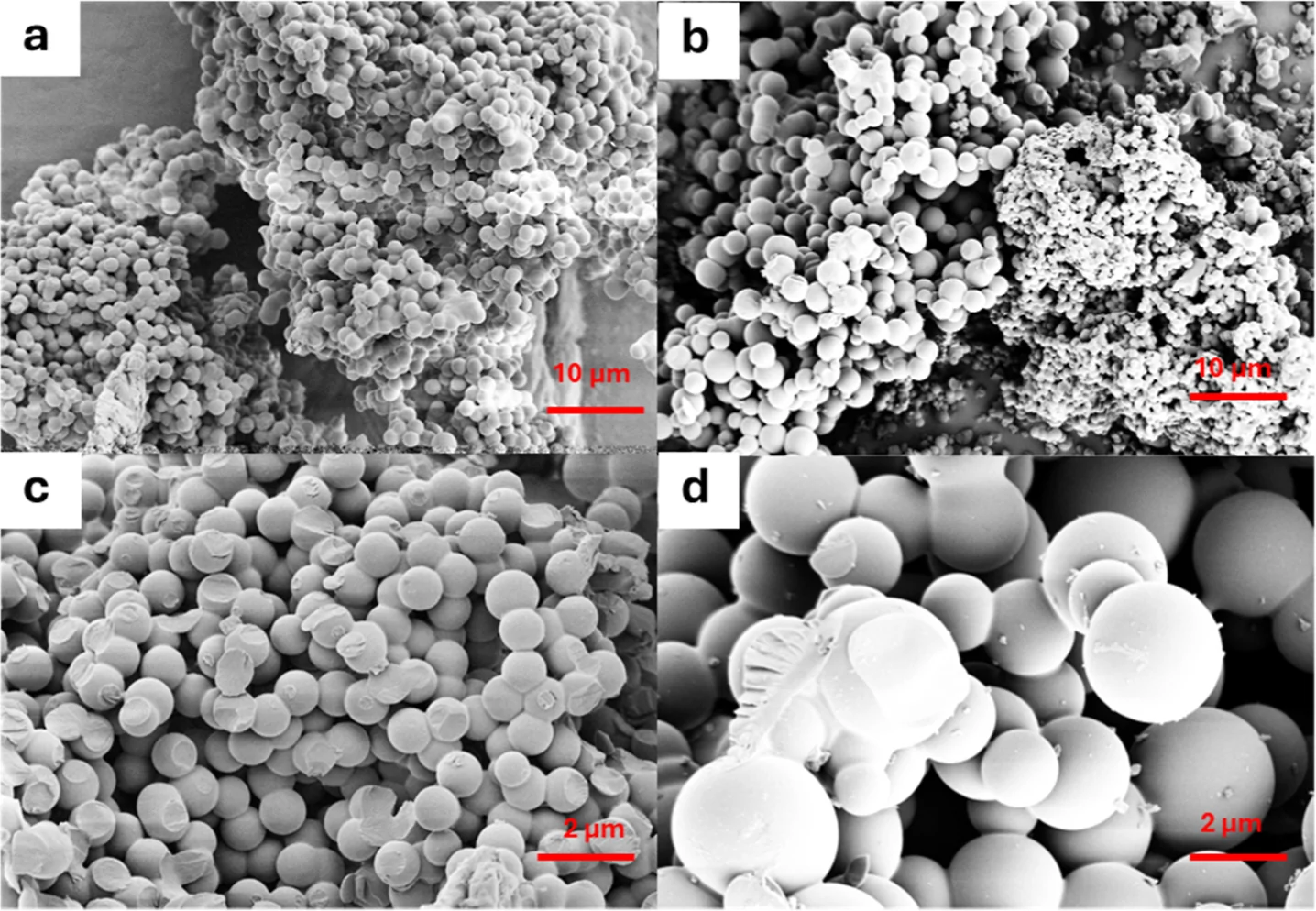
Scanning electron microscopy (SEM) images acquired in secondary electron mode (SE2 detector). The accelerating voltage (EHT) was set to 2.00 kV for Pyro-HC (a, c) and 3.00 kV for HC (b, d).

**Figure 7:**
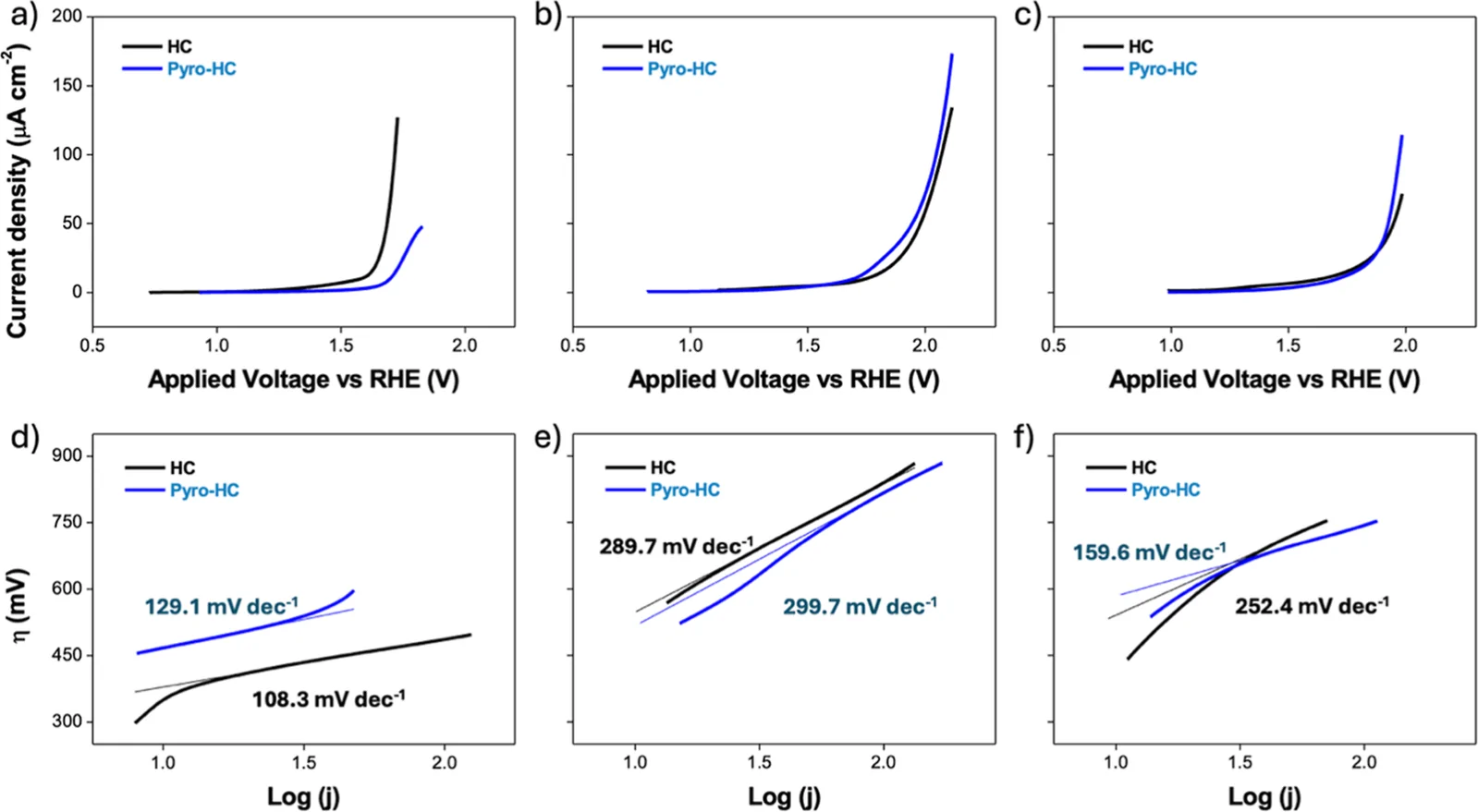
(a, b, c) LSV curves of HC and Pyro-HC electrodes, in alkaline, neutral, and acidic solutions, respectively. (d, e, f) Corresponding Tafel plots and fit of the linear region.

To place the selected materials in context, a comparison with selected glucose-derived hydrochars as reported in the literature is provided in Table S5. The comparison shows that the present material is consistent with the expected spherical morphology and chemistry of carbohydrate-derived hydrochars, while the main contribution of this work lies in the systematic evaluation of HTC conditions, structural evolution, changes due to pyrolytic post-treatment, and the electrochemical response within a unified experimental framework.

### 3.5 Electrochemical Characterization

Electrochemical measurements were conducted to probe the interfacial charge storage properties of HC and Pyro-HC in different electrolyte environments. The electrochemical response was used as a tool to evaluate surface accessibility and interfacial stability rather than to assess the catalytic performance. Prior to measurements, hydrochar films were prepared by drop-casting onto glassy carbon (GC) substrates to ensure uniform electrode surfaces. Cyclic voltammetry (CV) was first performed at 50 mV s^–1^ as a conditioning step to stabilize the electrode surface. Subsequent CV measurements were conducted at scan rates, from 50 to 10 mV s^–1^, to evaluate double-layer capacitance (Cdl) and electrochemically accessible interfacial charge storage behavior, as shown in Figures S8 and S9. Charge storage values were obtained by integrating the CV current response, and the corresponding capacitance values are summarized in Table 3 and Figure S10.

**Table 3:** Double-Layer Capacitance (C_dl_) and Integrated Charge (Q) of Both Materials, Measured at Different pH Values^a^

	HC			
pH	C_dl_ (μF cm^–2^)	Q (μC cm^–2^)	Tafel slope (mV dec^–1^)	J (μA cm^–2^) @ 1.7 V vs RHE
alkaline	66 ± 1	26.2	108.3 ± 0.4	70.1
neutral	66 ± 2	13.6	289.7 ± 0.1	8.0
acidic	60 ± 1	10.5	252.4 ± 1.0	12.4
	**Pyro-HC**			
alkaline	79	15.5	129.1 ± 0.2	10.7
neutral	205	41.2	299.8 ± 0.6	10.3
acidic	106	16.2	159.6 ± 0.6	9.5

a Bulk Cdl values were obtained from CVs in the non-Faradaic region, and Q represents the charge associative with the surface electrochemical activity.

The electrical double-layer capacitance (Cdl) and integrated charge were evaluated as a function of electrolyte pH to distinguish interfacial accessibility effects from Faradaic contributions. The Cdl values for HC remained relatively stable across the investigated pH range, varying between approximately 60 and 66 μF cm^–2^. In contrast, Pyro-HC exhibited moderate variations in Cdl from 79 to 205 μF cm^–2^ across different pHs. The capacitances obtained are in good agreement with literature values for smooth-surface carbon materials, making them ideal model systems for kinetic investigations.^37,55,56^ This behavior can be attributed to increased sp^2^ carbon and the moieties and nature of the surface oxygen functional group, which may facilitate interfacial charge distribution and surface morphology.^55–57^ This suggests that thermal treatment modifies surface chemistry and interfacial wettability instead of originating from changes in the particle morphology or microsphere size. However, the relatively low integrated charge values indicate that charge storage is predominantly governed by non-Faradaic double-layer charging, with minimal pseudocapacitive contribution.^58,59^ Linear sweep voltammetry (LSV) was performed at 2 mV s^–1^ to evaluate oxygen evolution reaction (OER) kinetics and to use OER as a probe reaction for surface chemistry effects rather than catalytic benchmark. All potentials were corrected for 80% solution resistance and referenced to the reversible hydrogen electrode (RHE) to enable reliable comparison between electrolytes.

The OER activity of both materials was dependent on electrolyte pH. Under alkaline conditions, Tafel slopes of approximately 108 mV dec^–1^ were obtained, indicating relatively faster reaction kinetics compared to neutral and acidic media, likely due to the favorable adsorption of hydroxyl intermediates on oxygen functionalized carbon surfaces, which may facilitate electron transfer and O–O bond formation. In the neutral electrolyte, Tafel slopes exceeded 200 mV dec^–1^, indicating sluggish reaction kinetics associated with limited availability of reactive surface chemistry. Hence, the OER activity of both HC and Pyro-HC remains characteristic of metal-free carbon electrodes and is primarily governed by surface oxygen chemistry rather than intrinsic catalytic activity.^60^ As shown in Figure S11a, Pyro-HC shows larger currents than the glassy carbon, which does not even reach 10 μA cm^–2^ at 1.75 V vs RHE. This proves that the observed activity originates solely from carbonaceous powder. Stability tests were performed via CVs with fresh electrodes and after OER tests. Minimal variation in electrochemical response was observed, indicating good structural and interfacial stability of the hydrochar films on the electrode substrate. Additionally, chronoamperometric measurements performed under constant potential showed stable current responses over 1 h (Figure S11b), further confirming the structural and electrochemical robustness of the materials under operational conditions. The kinetic results are similar to carbon materials with limited reactive surface chemistry, and a comparison is reported in Table S4.^61^

## 4 Conclusion

This study employed hydrothermal carbonization of glucose to produce hydrochars and systematically investigated carbon formation pathways as a function of reaction severity. The combined analysis of solid, liquid, and gaseous carbon fractions demonstrates that increasing the temperature and residence time promotes dehydration, decarboxylation, and aromatization reactions, leading to progressive carbon densification and structural ordering. Van Krevelen analysis provided quantitative evidence of oxygen elimination and structural condensation, establishing a direct link between reaction parameters and the chemical evolution of the carbon matrix. Post-thermal treatment further enhanced structural organization and reduced surface oxygen functionality, promoting the formation of more conjugated sp^2^-rich carbon domains without significantly altering the overall spherical morphology. Electrochemical characterization revealed that charge storage behavior is primarily governed by surface chemistry and interfacial accessibility rather than morphological textural features. The relatively constant double-layer capacitance of pristine hydrochar across different electrolyte pH values indicates a stable electrolyte wetting and interfacial charge distribution. In contrast, pyrolyzed hydrochar exhibited moderately enhanced capacitance and charge density, from reduced oxygen-containing groups and increased aromatic carbon character. Nevertheless, charge storage remained dominated by electrostatic double-layer processes, consistent with metal-free carbon electrode behavior. OER studies confirmed intrinsically sluggish catalytic kinetics typical of carbon-based electrodes, with slightly improved kinetics in alkaline media compared to those in neutral and acidic environments. Long-term chronoamperometric measurements further confirmed the excellent electrochemical stability and structural robustness of the hydrochar-based electrodes. These findings demonstrate the potential of these materials for electrochemical applications as carbon supports and as platforms for functionalization in processes occurring at the electrode–electrolyte interface in energy conversion and storage systems. Thus, this study establishes a reproducible framework for understanding hydrothermal carbon formation through quantitative correlations among reaction severity, carbon partitioning, structural evolution, and interfacial electrochemical behavior, positioning hydrochars as model biomass-derived carbon materials for fundamental studies in electrochemistry. These results provide guidance for the rational design of sustainable biomass-derived carbon materials for value-added applications.

## Supplementary Material



## Notes

HTC sample labels, hydrochar preparation and pyrolysis schemes, solid/gas/liquid yield calculations, atomic H/C and O/C ratio calculations, electrode-preparation scheme, Van Krevelen analysis, FTIR spectra and band assignments, Raman spectra before background correction, cyclic voltammetry curves, charging-current analysis, non-Faradaic charge and capacitance comparison, LSV comparison with glassy carbon, chronoamperometric stability test, electrochemical performance comparison with reference carbon materials, and supporting references (PDF)

## References

[1] Varma R. S. (2019). Biomass-Derived
Renewable Carbonaceous Materials for Sustainable Chemical and Environmental
Applications. ACS Sustainable Chem. Eng..

[2] Kang S., Li X., Fan J., Chang J. (2012). Characterization of Hydrochars Produced
by Hydrothermal Carbonization of Lignin, Cellulose, d-Xylose, and
Wood Meal. Ind. Eng. Chem. Res..

[3] Wortmann M., Keil W., Brockhagen B., Biedinger J., Westphal M., Weinberger C., Diestelhorst E., Hachmann W., Zhao Y., Tiemann M., Reiss G., Hüsgen B., Schmidt C., Sattler K., Frese N. (2022). Pyrolysis
of sucrose-derived hydrochar. J. Anal. Appl.
Pyrolysis.

[4] Yuan S., Brown A., Zheng Z., Johnson R. L., Agro K., Kruse A., Timko M. T., Schmidt-Rohr K. (2024). Glucose hydrochar
consists of linked phenol, furan, arene, alkyl, and ketone structures
revealed by advanced solid-state nuclear magnetic resonance. Solid State Nucl. Magn. Reson..

[5] Titirici M.-M., Antonietti M. (2010). Chemistry
and materials options of sustainable carbon
materials made by hydrothermal carbonization. Chem. Soc. Rev..

[6] Sevilla M., Fuertes A. B. (2009). The production of
carbon materials by hydrothermal
carbonization of cellulose. Carbon N. Y..

[7] Ischia G., Cutillo M., Guella G., Bazzanella N., Cazzanelli M., Orlandi M., Miotello A., Fiori L. (2022). Hydrothermal
carbonization of glucose: Secondary char properties, reaction pathways,
and kinetics. Chemical Engineering Journal.

[8] Abdeldayem O. M., Dupont C., Ferras D., Kennedy M. (2025). An experimental
and
numerical investigation of secondary char formation in hydrothermal
carbonization: revealing morphological changes via hydrodynamics. RSC Adv..

[9] Sevilla M., Fuertes A. B. (2009). Chemical and Structural
Properties of Carbonaceous
Products Obtained by Hydrothermal Carbonization of Saccharides. Chem.—Eur. J..

[10] Shi N., Liu Q., He X., Wang G., Chen N., Peng J., Ma L. (2019). Molecular
Structure and Formation Mechanism of Hydrochar from Hydrothermal
Carbonization of Carbohydrates. Energy Fuels.

[11] Higgins L. J. R., Brown A. P., Harrington J. P., Ross A. B., Kaulich B., Mishra B. (2020). Evidence for a core-shell
structure of hydrothermal
carbon. Carbon N. Y..

[12] Elaigwu S. E., Greenway G. M. (2016). Chemical, structural and energy properties
of hydrochars
from microwave-assisted hydrothermal carbonization of glucose. Int. J. Ind. Chem..

[13] Guo S., Dong X., Wu T., Zhu C. (2016). Influence of reaction
conditions and feedstock on hydrochar properties. Energy Convers. Manage..

[14] Fechler N., Wohlgemuth S.-A., Jäker P., Antonietti M. (2013). Salt and sugar:
direct synthesis of high surface area carbon materials at low temperatures
via hydrothermal carbonization of glucose under hypersaline conditions. J. Mater. Chem. A.

[15] Zhuang X., Liu J., Ma L. (2023). Facile synthesis
of hydrochar-supported catalysts from
glucose and its catalytic activity towards the production of functional
amines. Green Energy Environ..

[16] Khosravi A., Zheng H., Liu Q., Hashemi M., Tang Y., Xing B. (2022). Production and characterization
of hydrochars and their application
in soil improvement and environmental remediation. Chem. Eng. J..

[17] Prieto M., Yue H., Brun N., Ellis G. J., Naffakh M., Shuttleworth P. S. (2024). Hydrothermal
Carbonization of Biomass for Electrochemical Energy Storage: Parameters,
Mechanisms, Electrochemical Performance, and the Incorporation of
Transition Metal Dichalcogenide Nanoparticles. Polymers (Basel).

[18] Espro C., Satira A., Mauriello F., Anajafi Z., Moulaee K., Iannazzo D., Neri G. (2021). Orange peels-derived
hydrochar for
chemical sensing applications. Sens. Actuators,
B.

[19] Qian Y., Li Y., Pan Z., Tian J., Lin N., Qian Y. (2021). Hydrothermal
“Disproportionation” of Biomass into Oriented Carbon
Microsphere Anode and 3D Porous Carbon Cathode for Potassium Ion Hybrid
Capacitor. Adv. Funct. Mater..

[20] Gao Y., Liu C., Jiang Y., Zhang Y., Wei Y., Zhao G., Liu R., Liu Y., Shi G., Wang G. (2024). Hydrothermal assisting
biomass into a porous active carbon for high-performance supercapacitors. Diamond Relat. Mater..

[21] Yan B., Han C., Dai Y., Li M., Wu Z., Gao X. (2024). Biomass derived
hard carbon materials for sodium ion battery anodes: Exploring the
influence of carbon source on structure and sodium storage performance. Fuel.

[22] Tovar-Martinez E., Sanchez-Rodriguez C. E., Sanchez-Vasquez J. D., Reyes-Reyes M., López-Sandoval R. (2023). Synthesis
of carbon spheres from
glucose using the hydrothermal carbonization method for the fabrication
of EDLCs. Diamond Relat. Mater..

[23] Thulluru L. P., Dhanda A., Doki M. M., Ghangrekar M. M., Chowdhury S. (2025). Sludge-derived hydrochar as a potential electrocatalyst
for improved CO2 reduction in microbial electrosynthesis. RSC Sustainability.

[24] Peng J., Kang X., Zhao S., Zhao P., Ragauskas A. J., Si C., Xu T., Song X. (2023). Growth mechanism of glucose-based
hydrochar under the effects of acid and temperature regulation. J. Colloid Interface Sci..

[25] Lin Y., Xu H., Gao Y., Zhang X. (2023). Preparation and characterization
of hydrochar-derived activated carbon from glucose by hydrothermal
carbonization. Biomass Convers. Biorefin..

[26] He Q., Yu Y., Wang J., Suo X., Liu Y. (2021). Kinetic Study of the
Hydrothermal Carbonization Reaction of Glucose and Its Product Structures. Ind. Eng. Chem. Res..

[27] Qiu J., Huang B., Liu Y., Chen D., Xie Z. (2020). Glucose-derived
hydrothermal carbons as energy storage booster for vanadium redox
flow batteries. J. Energy Chem..

[28] Zhang X., Cao T., Zhang G., Liu Q., Kong G., Wang K., Jiang Y., Zhang X., Han L. (2024). Sustainable hydrothermal
carbon for advanced electrochemical energy storage. J. Mater. Chem. A.

[29] Magdziarz A., Wilk M., Wądrzyk M. (2020). Pyrolysis of hydrochar derived from
biomass – Experimental investigation. Fuel.

[30] Trivedi P., Minj N. C., Mittal S., Kamaraj B., Yadav S., Sengeni A. (2025). How common is it to
get an OER overpotential that is
<250 mV?. J. Mater. Chem. A.

[31] Fabbri E., Schmidt T. J. (2018). Oxygen Evolution Reaction - The Enigma in Water Electrolysis. ACS Catal..

[32] Shi Y., Xie R., Liu X., Zhang N., Aruta C., Yang N. (2019). Tunable pH-dependent
oxygen evolution activity of strontium cobaltite thin films for electrochemical
water splitting. Phys. Chem. Chem. Phys..

[33] Zeng H., Chen J., Wang C., Qi J., Liu Z., Li M., Gu L., Wang J., Hong E., Zhang Y., Xu J., Yang C. (2024). Understanding the pH-Dependent Catalytic Activity for
the Layered LixCoO2 Oxygen Evolution Catalysts. ACS Mater. Lett..

[34] Fiori L., Basso D., Castello D., Baratieri M. (2014). Hydrothermal
Carbonization of Biomass: Design of a Batch Reactor and Preliminary
Experimental Results. Chem. Eng. Trans..

[35] Burguete P., Corma A., Hitzl M., Modrego R., Ponce E., Renz M. (2016). Fuel and chemicals from wet lignocellulosic biomass waste streams
by hydrothermal carbonization. Green Chem..

[36] Basso D., Weiss-Hortala E., Patuzzi F., Baratieri M., Fiori L. (2018). In Deep Analysis on the Behavior of Grape Marc Constituents during
Hydrothermal Carbonization. Energies (Basel).

[37] Costa
de Oliveira M. A., Schröder C., Brunet Cabré M., Nolan H., Forner-Cuenca A., Perova T. S., McKelvey K., Colavita P. E. (2024). Effects of N-functional groups on the electron transfer
kinetics of VO2+/VO2+ at carbon: Decoupling morphology from chemical
effects using model systems. Electrochim. Acta.

[38] Knežević D., Van Swaaij W. P. M., Kersten S. R. A. (2009). Hydrothermal Conversion of Biomass:
I, Glucose Conversion in Hot Compressed Water. Ind. Eng. Chem. Res..

[39] Marzban N., Libra J. A., Rotter V. S., Ro K. S., Moloeznik
Paniagua D., Filonenko S. (2023). Changes in Selected Organic and Inorganic
Compounds in the Hydrothermal Carbonization Process Liquid While in
Storage. ACS Omega.

[40] Śliz M., Wilk M. (2020). A comprehensive investigation of
hydrothermal carbonization: Energy
potential of hydrochar derived from Virginia mallow. Renewable Energy.

[41] Alcazar-Ruiz A., Villardon A., Dorado F., Sanchez-Silva L. (2023). Hydrothermal
carbonization coupled with fast pyrolysis of almond shells: Valorization
and production of valuable chemicals. Waste
Manage..

[42] van
Zandvoort I., Wang Y., Rasrendra C. B., van Eck E. R. H., Bruijnincx P. C. A., Heeres H. J., Weckhuysen B. M. (2013). Formation,
Molecular Structure, and Morphology of Humins in Biomass Conversion:
Influence of Feedstock and Processing Conditions. ChemSusChem.

[43] Li J., Zhao P., Li T., Lei M., Yan W., Ge S. (2020). Pyrolysis behavior of hydrochar from
hydrothermal carbonization of
pinewood sawdust. J. Anal. Appl. Pyrolysis.

[44] Saha N., Reza M. T. (2021). Effect of pyrolysis on basic functional
groups of hydrochars. Biomass Convers. Biorefin..

[45] Acosta A. C., Arias C. A., Biller P., Wittig N. K., Baragau I.-A., Alhnidi M. J., Ravenni G., Sárossy Z., Benedini L., Abramiuc L. E., Popescu D.-G., Negassa W., Marulanda V. F., Müller-Stöver D. S., Brix H. (2024). Hydrothermal carbonization and pyrolysis in wetland engineering:
Carbon sequestration, phosphorus recovery, and structural characterization
of willow-based chars with X-ray μ-computed tomography. Chem. Eng. J..

[46] Lesiak B., Kövér L., Tóth J., Zemek J., Jiricek P., Kromka A., Rangam N. (2018). C sp2/sp3 hybridisations in carbon
nanomaterials – XPS and (X)­AES study. Appl. Surf. Sci..

[47] Teng C.-C., Ma C.-C. M., Lu C.-H., Yang S.-Y., Lee S.-H., Hsiao M.-C., Yen M.-Y., Chiou K.-C., Lee T.-M. (2011). Thermal
conductivity and structure of non-covalent functionalized graphene/epoxy
composites. Carbon N. Y..

[48] Smith M., Scudiero L., Espinal J., McEwen J.-S., Garcia-Perez M. (2016). Improving
the deconvolution and interpretation of XPS spectra from chars by
ab initio calculations. Carbon N. Y..

[49] Peruzzi C., Battistoni S., Montesarchio D., Cocuzza M., Marasso S. L., Verna A., Pasquardini L., Verucchi R., Aversa L., Erokhin V., D’Angelo P., Iannotta S. (2021). Interfacing aptamers,
nanoparticles and graphene in a hierarchical structure for highly
selective detection of biomolecules in OECT devices. Sci. Rep..

[50] Lascovich J. C., Giorgi R., Scaglione S. (1991). Evaluation
of the sp2/sp3 ratio in
amorphous carbon structure by XPS and XAES. Appl. Surf. Sci..

[51] Lesiak B., Zemek J., Jiricek P., Malolepszy A., Stobinski L. (2017). Influence of the preparation conditions
of Pd-ZrO2
and AuPd-ZrO2 nanoparticle–decorated functionalised MWCNTs:
Electron spectroscopy study aided with the QUASES. Surf. Interface Anal..

[52] Ferrari A.
C., Robertson J. (2000). Interpretation
of Raman spectra of disordered and amorphous
carbon. Phys. Rev. B.

[53] Wang Y., Alsmeyer D. C., McCreery R. L. (1990). Raman spectroscopy
of carbon materials:
structural basis of observed spectra. Chem.
Mater..

[54] Wortmann M., Keil W., Diestelhorst E., Westphal M., Haverkamp R., Brockhagen B., Biedinger J., Bondzio L., Weinberger C., Baier D., Tiemann M., Hütten A., Hellweg T., Reiss G., Schmidt C., Sattler K., Frese N. (2023). Hard carbon microspheres with bimodal size distribution and hierarchical
porosity via hydrothermal carbonization of trehalose. RSC Adv..

[55] Ranganathan S., Kuo T.-C., McCreery R. L. (1999). Facile
Preparation of Active Glassy
Carbon Electrodes with Activated Carbon and Organic Solvents. Anal. Chem..

[56] Hoque Md.K., Behan J. A., Stamatin S. N., Zen F., Perova T. S., Colavita P. E. (2019). Capacitive storage at nitrogen doped
amorphous carbon
electrodes: structural and chemical effects of nitrogen incorporation. RSC Adv..

[57] Andreas H. A., Conway B. E. (2006). Examination of the
double-layer capacitance of an high
specific-area C-cloth electrode as titrated from acidic to alkaline
pHs. Electrochim. Acta.

[58] Ji H., Zhao X., Qiao Z., Jung J., Zhu Y., Lu Y., Zhang L. L., MacDonald A. H., Ruoff R. S. (2014). Capacitance of carbon-based
electrical double-layer capacitors. Nat. Commun..

[59] Ge Y., Xie X., Roscher J., Holze R., Qu Q. (2020). How to measure and
report the capacity of electrochemical double layers, supercapacitors,
and their electrode materials. J. Solid State
Electrochem..

[60] Lu X., Yim W.-L., Suryanto B. H. R., Zhao C. (2015). Electrocatalytic Oxygen
Evolution at Surface-Oxidized Multiwall Carbon Nanotubes. J. Am. Chem. Soc..

[61] Costa
de Oliveira M. A., Zhang R., Schröder C., Pota F., Brunet Cabré M., McKelvey K., Colavita P. E. (2025). Synergistic
effects of surface chemistry and porosity in vanadium redox reactions:
from smooth thin films to high surface area carbon electrodes. Carbon N. Y..

