# Supplementary material for: Glucose-Derived Carbon Materials via Hydrothermal Carbonization and Pyrolysis for Electrochemical Applications
Source: ACS Omega. 2026 Sep 8;11(37):55820–30. doi: 10.1021/acsomega.6c05437 (PMC13613475; doi:10.1021/acsomega.6c05437)
Supplement: Supplementary file 1 [file ao6c05437_si_001.pdf]

Supporting Information

Glucose-Derived Carbon Materials via Hydrothermal Carbonization and Pyrolysis for Electrochemical Applications

Behnam Jabbari Kalkhoran, Alessandro Chesini, Maida A. Costa de Oliveira, Filippo Marchelli, Roberto Verucchi, Michele Orlandi, Luca Fiori*

Corresponding Author

Luca Fiori - Department of Civil, Environmental and Mechanical Engineering, University of Trento, Trento 38123, Italy; C3A-Center Agriculture Food Environment, University of Trento, Trento 38010, Italy; orcid.org/0000-0003-0585-3793; Email: luca.fiori@unitn.it

Authors

Behnam Jabbari Kalkhoran - C3A-Center Agriculture Food Environment, University of Trento, Trento 38010, Italy; orcid.org/0000-0001-9998-131X

Alessandro Chesini - Department of Physics, University of Trento, Trento 38123, Italy; orcid.org/0009-0004-3374-7407

Maida A. Costa de Oliveira - Department of Physics, University of Trento, Trento 38123, Italy; School of Chemistry, Trinity College Dublin, Dublin 2, Ireland; orcid.org/0000-0001-8234-9027

Filippo Marchelli - Department of Civil, Environmental and Mechanical Engineering, University of Trento, Trento 38123, Italy; Department of Civil, Chemical and Environmental Engineering, University of Genova, Genova 16145, Italy; orcid.org/0000-0003-4189-391X

Roberto Verucchi - CNR-IMEM, Trento unit c/o FBK, Italy; orcid.org/0000-0001-6850-3980

Michele Orlandi - - Department of Physics, University of Trento, Trento 38123, Italy;orcid.org/0000-0001-5738-3231

Table S1. Labelled samples from HTC synthesis.

Temperature (°C)	Time (hour)	B/W	Labelled samples
180	0.5	0.25	180C-0.5h-B/W=0.25
180	0.5	0.5	180C-0.5h-B/W=0.5
180	0.5	1.0	180C-0.5h-B/W=1
180	3.0	0.25	180C-3h-B/W=0.25
180	3.0	0.5	180C-3h-B/W=0.5
180	3.0	1.0	180C-3h-B/W=1
210	0.5	0.25	210C-0.5h-B/W=0.25
210	0.5	0.5	210C-0.5h-B/W=0.5
210	0.5	1.0	210C-0.5h-B/W=1
210	3.0	0.25	210C-3h-B/W=0.25
210	3.0	0.5	210C-3h-B/W=0.5
210	3.0	1.0	210C-3h-B/W=1

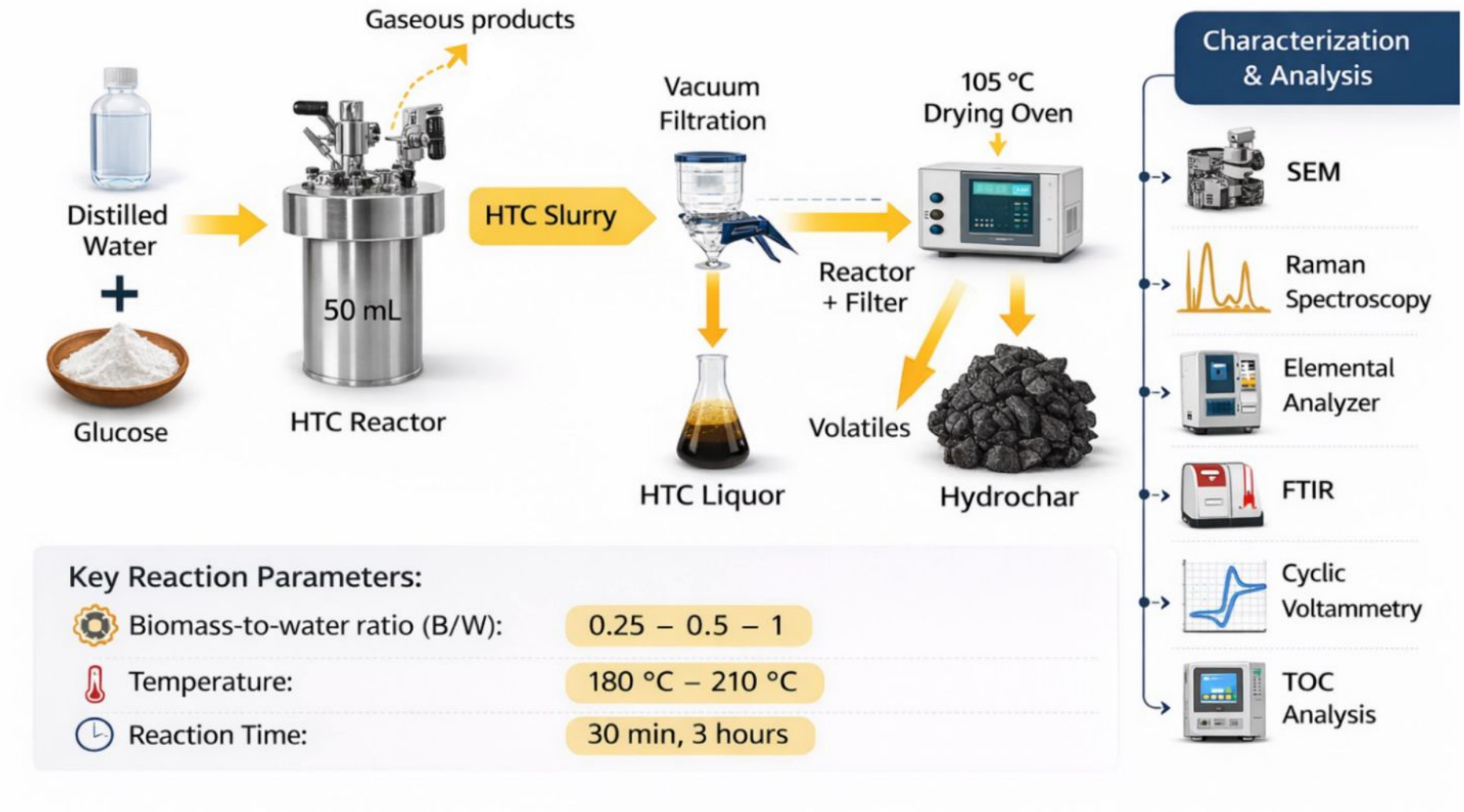

Figure S1. Schematic of hydrochar preparation and sample labelling.

***Yield Calculations.* The solid yield (SY), gaseous yield (GY), and liquid yield (LY) were calculated based on the initial glucose mass. SY was determined from the dried hydrochar mass using the equation:**

$$SY(\%) = \left(\frac{Mass\ of\ dry\ hydrochar}{Initial\ mass\ of\ dry\ glucose}\right) \times 100 \qquad \text{eq. S1}$$

GY was calculated based on the volume of gaseous products, using the ideal gas law to estimate the mass of gas produced:

$$GY(\%) = \left(\frac{Mass\ of\ gas\ product}{Initial\ mass\ of\ dry\ glucose}\right) \times 100 \qquad \text{eq. S2}$$

The liquid yield (LY) was determined by difference:

$$LY\ (\%) = 100 - SY\ (\%) - GY\ (\%) \qquad \text{eq. S3}$$

Table S2. HTC yields under 3 replicates.

**Condition**	**SY %**		**GY %**		**LY %**
	**(average)**	(+/-)	**(average)**	(+/-)	(Calculated)
180C-0.5h-B/W=0.25	10.3	0.7	0.3	0.1	89.4
180C-0.5h-B/W=0.5	2.6	0.7	0.2	0.0	97.2
180C-0.5h-B/W=1	19.1	1.3	0.3	0.0	80.6
180C-3h-B/W=0.25	18.1	1.4	0.9	0.2	81.0
180C-3h-B/W=0.5	27.1	2.2	0.8	0.1	72.1
180C-3h-B/W=1	63.1	1.7	0.9	0.0	36.0
210C-0.5h-B/W=0.25	26.8	3.3	1.4	0.1	71.8
210C-0.5h-B/W=0.5	25.1	3.0	1.1	0.5	73.8
210C-0.5h-B/W=1	61.3	2.3	1.8	0.3	36.9
210C-3h-B/W=0.25	44.6	1.7	4.4	0.3	51.0
210C-3h-B/W=0.5	46.9	2.0	5.2	0.3	47.9
210C-3h-B/W=1	50.2	4.6	4.9	1.9	45.6

The gaseous yield (GY) was mostly influenced by temperature and residence time. At 180 °C, GY was approximately 0.3% after 0.5 h and increased to 0.9% after 3 h. Higher temperatures and longer residence times generally led to higher GY, with the maximum observed at 210 °C, 0.5 h, and B/W=0.5, reaching 5.2%. Similar values were observed for 210 °C, 0.5 h, at B/W=1

(4.9%) and B/W=0.25 (4.4%), consistently with the expected trend that increased thermal degradation promotes gas formation.

The atomic ratios of hydrogen and oxygen relative to carbon were calculated as shown in Equations S4 and S5. In Figure S4, H, O, and C represent hydrogen, oxygen, and carbon, respectively. MW denotes the molar mass of H (1.008 g mol⁻¹), O (16.00 g mol⁻¹), and C (12.01 g mol⁻¹). The oxygen content was determined as described in the Materials and Methods section.

$$H/C = \frac{H\,(\%)/MW_H}{C\,(\%)/MW_C} \qquad \text{eq. S4}$$

$$O/C = \frac{O\,(\%)/MW_O}{C\,(\%)/MW_C} \qquad \text{eq. S5}$$

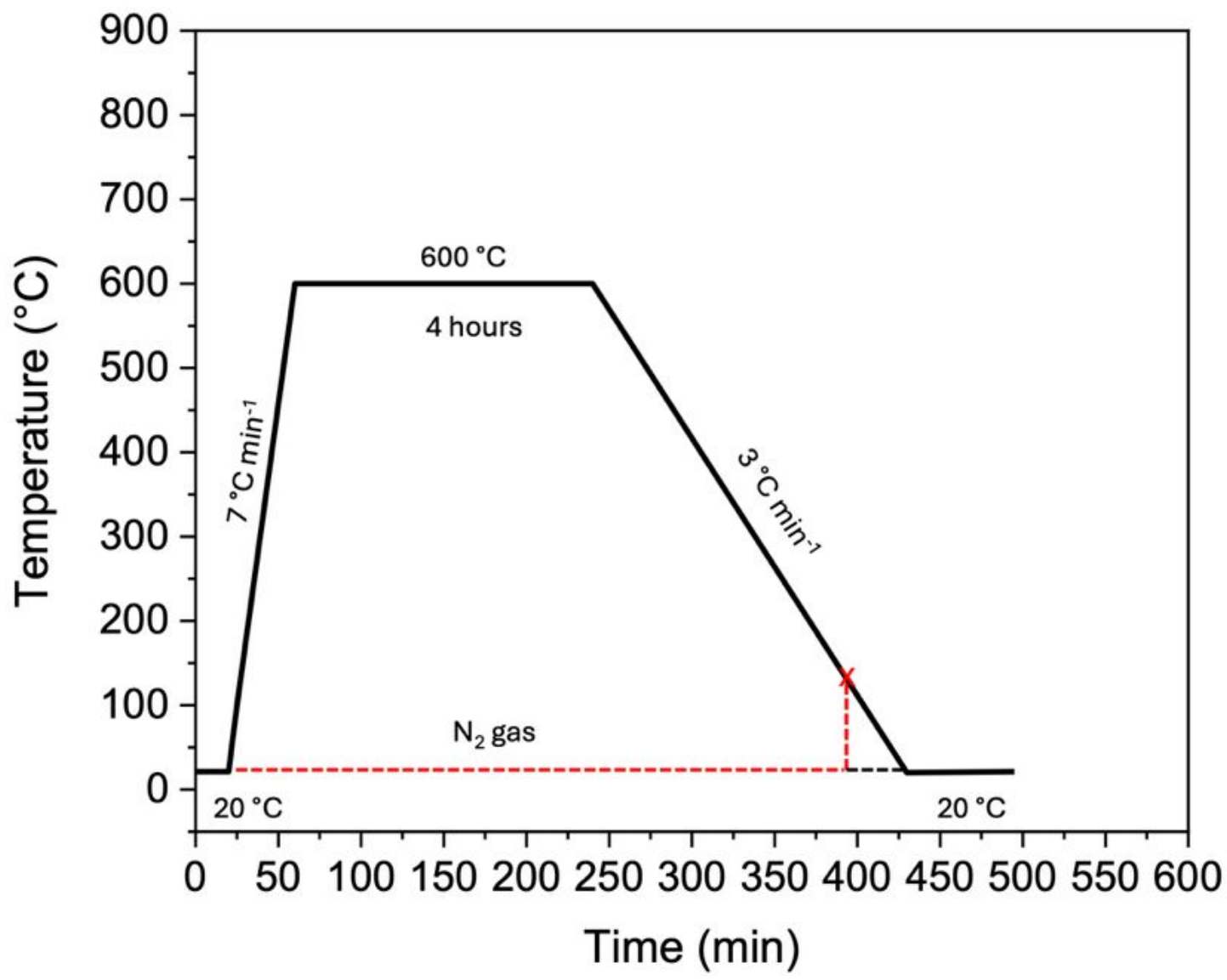

Figure S2. Conditions used for pyrolysis of hydrochar.

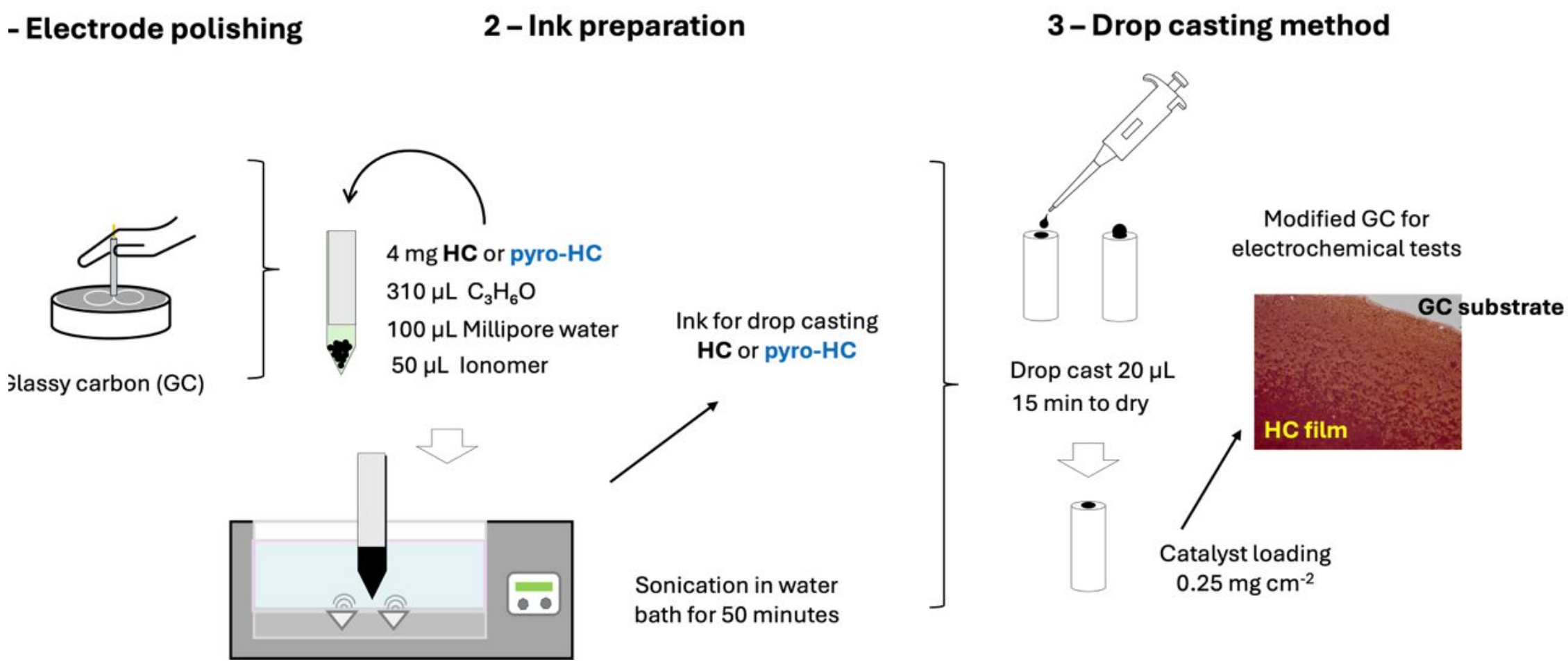

Figure S3. Schematic of electrode preparation for electrochemical studies.

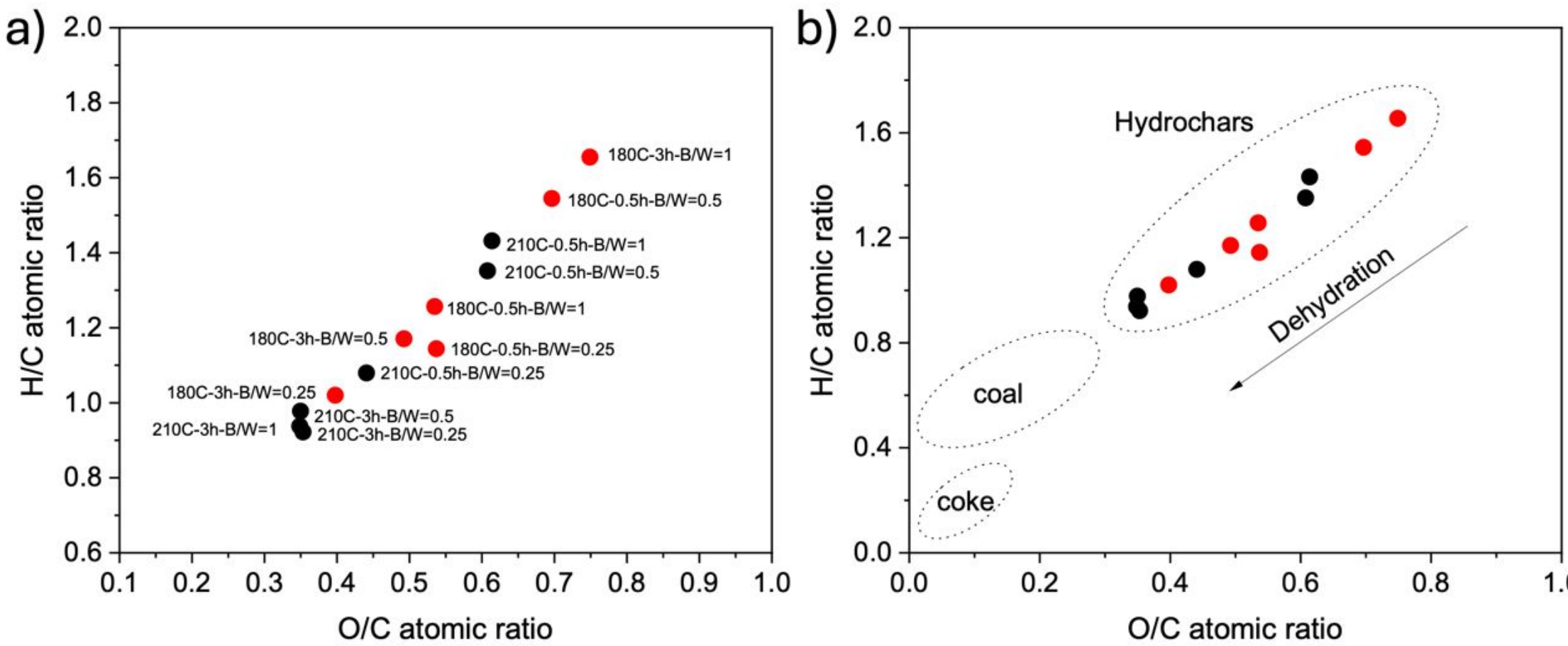

Figure S4. Van Krevelen diagram showing H/C and O/C ratios of hydrochars prepared under selected conditions. The red and black indicators refer to samples obtained at 180 and 210 °C, respectively. (a) graph with a detailed indication of all samples; (b) graph showing the same samples with an indication of the areas of relevance of the hydrochars – present study – coal, and coke and of the main reaction occurring during HTC: dehydration.

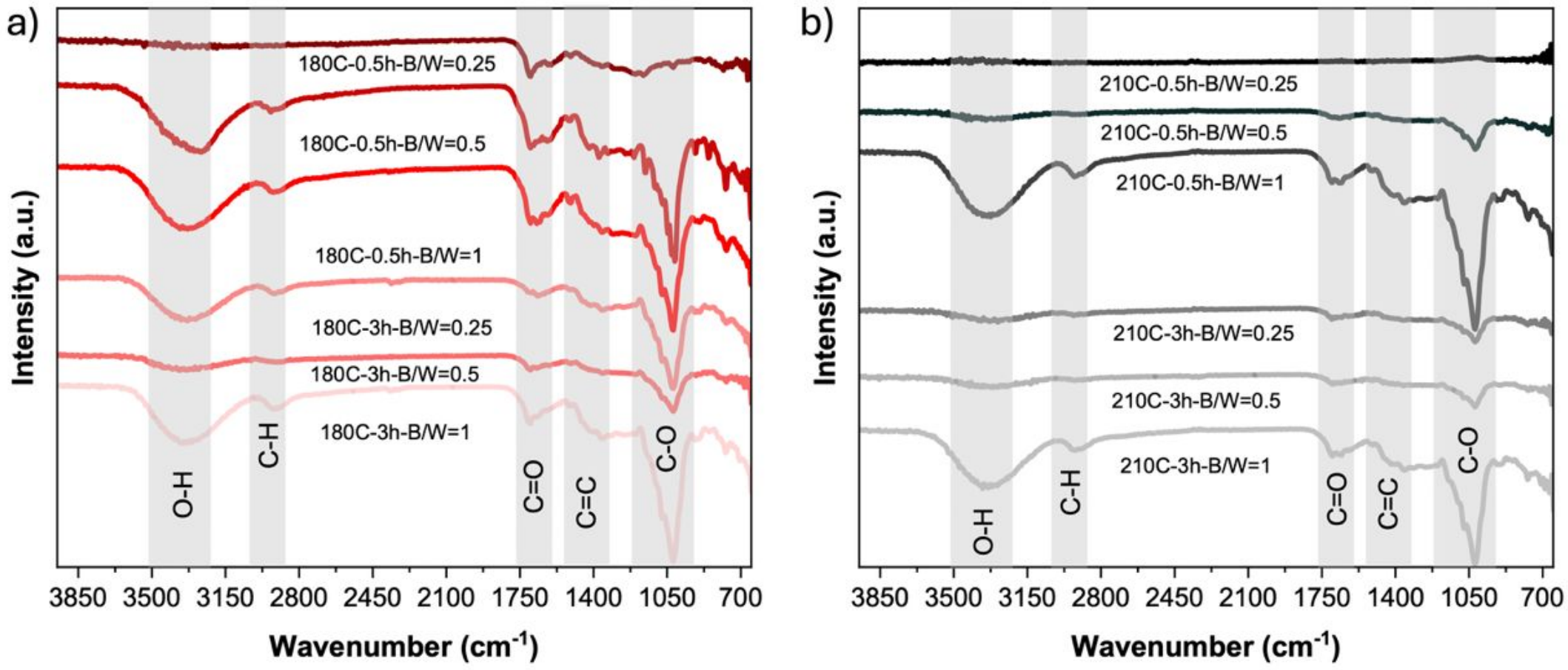

Figure S5. FTIR spectra of hydrochar samples: (a) samples obtained at 180 °C; (b) samples obtained at 210 °C.

Table S3. FTIR band assignments for hydrochar samples.

Mode assignments	Peak positions (cm^{-1})	References
O–H stretching (hydroxyl / hydrogen-bonded hydroxyls; sometimes residual moisture)	3700 - 3000; 3430 -3400	[1–4]
Aliphatic C–H stretching	2930 - 2930;3000 -2815	[1,3,4]
C=O stretching (carbonyl / quinone / ester / carboxyl; keto-aliphatic linkages)	1710 - 1695; in some glucose-based hydrochars up to 1760	[2–5]
Aromatic C=C ring stretching	1620 - 1595	[2,3,5]
Additional aromatic ring band	1513 - 1510	[2,5]
O–H deformation / bending	1360; 1290	[2]
C–O stretching / O–H bending (hydroxyl, ester, ether)	1000 - 1450	[1,5]
C–O stretching	1030; 1015	[2,3]
C–O–C ether stretching (furanic ring structures	1150	[2]
Aromatic C–H out-of-plane bending	875 -750; often 800 or 780	[1,2,5]

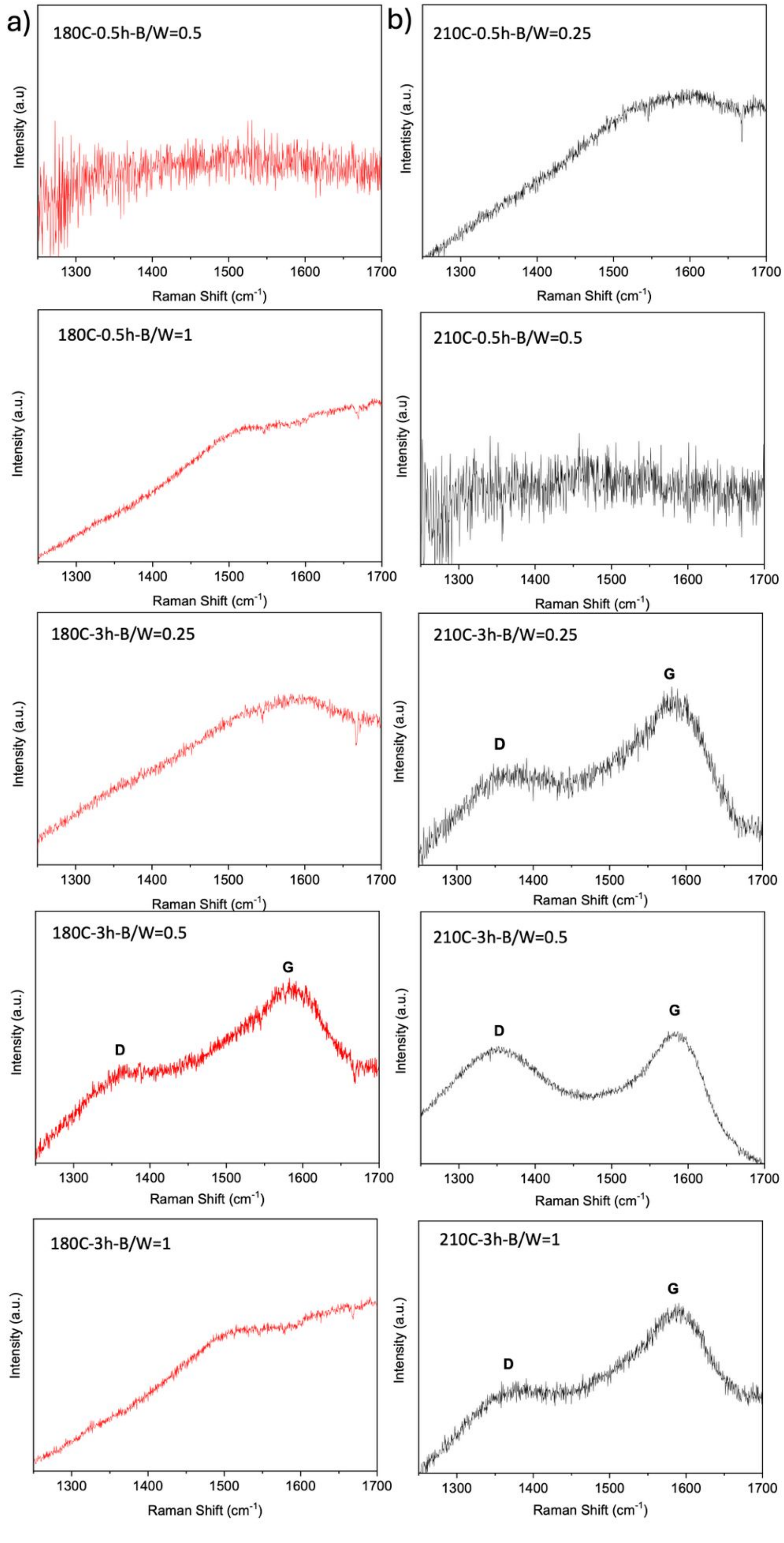

Figure S6. Raman spectra of hydrochars without background correction: (a) and underlying: samples obtained at 180 °C; (b) and underlying: samples obtained at 210 °C.

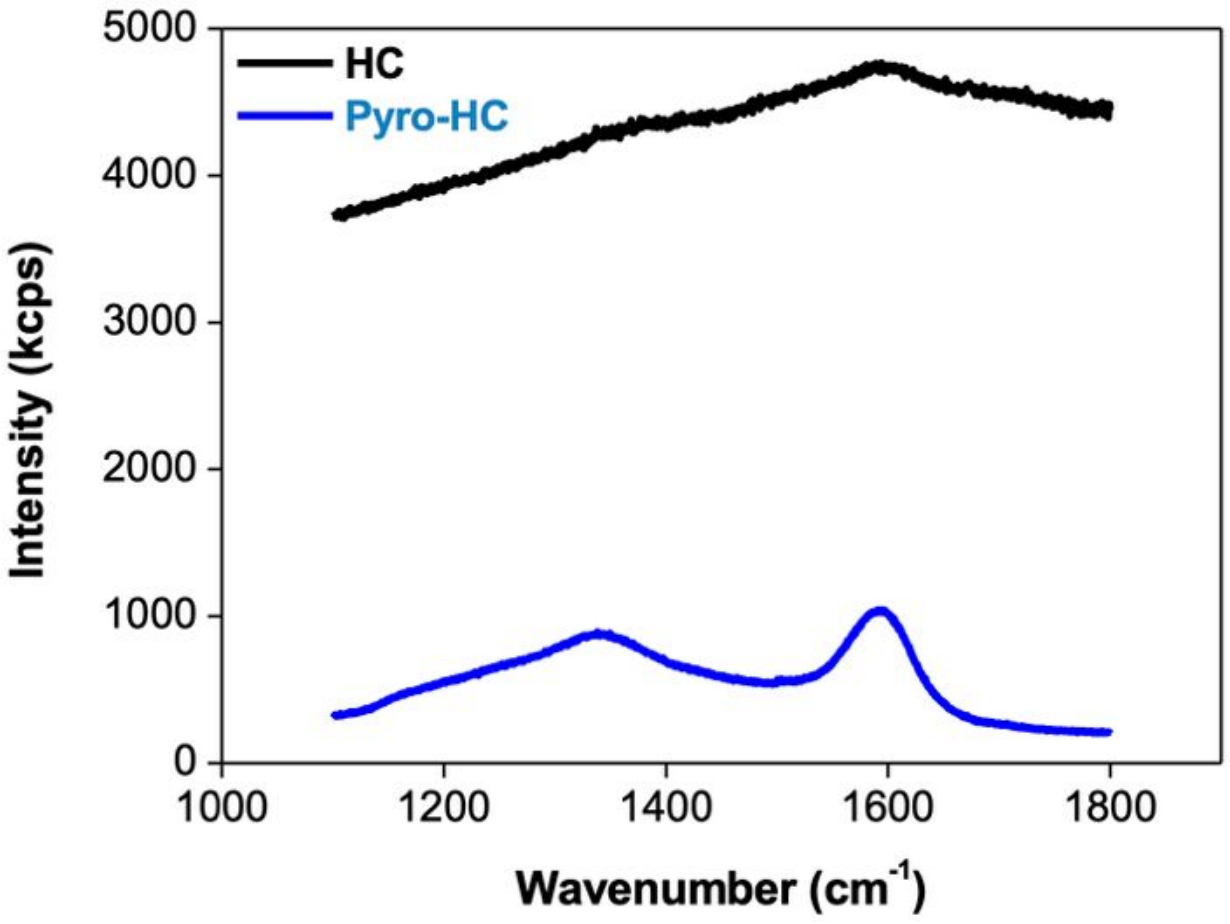

Figure S7. Raman spectra of HC and Pyro-HC without background subtraction, showing the large background contribution in HC.

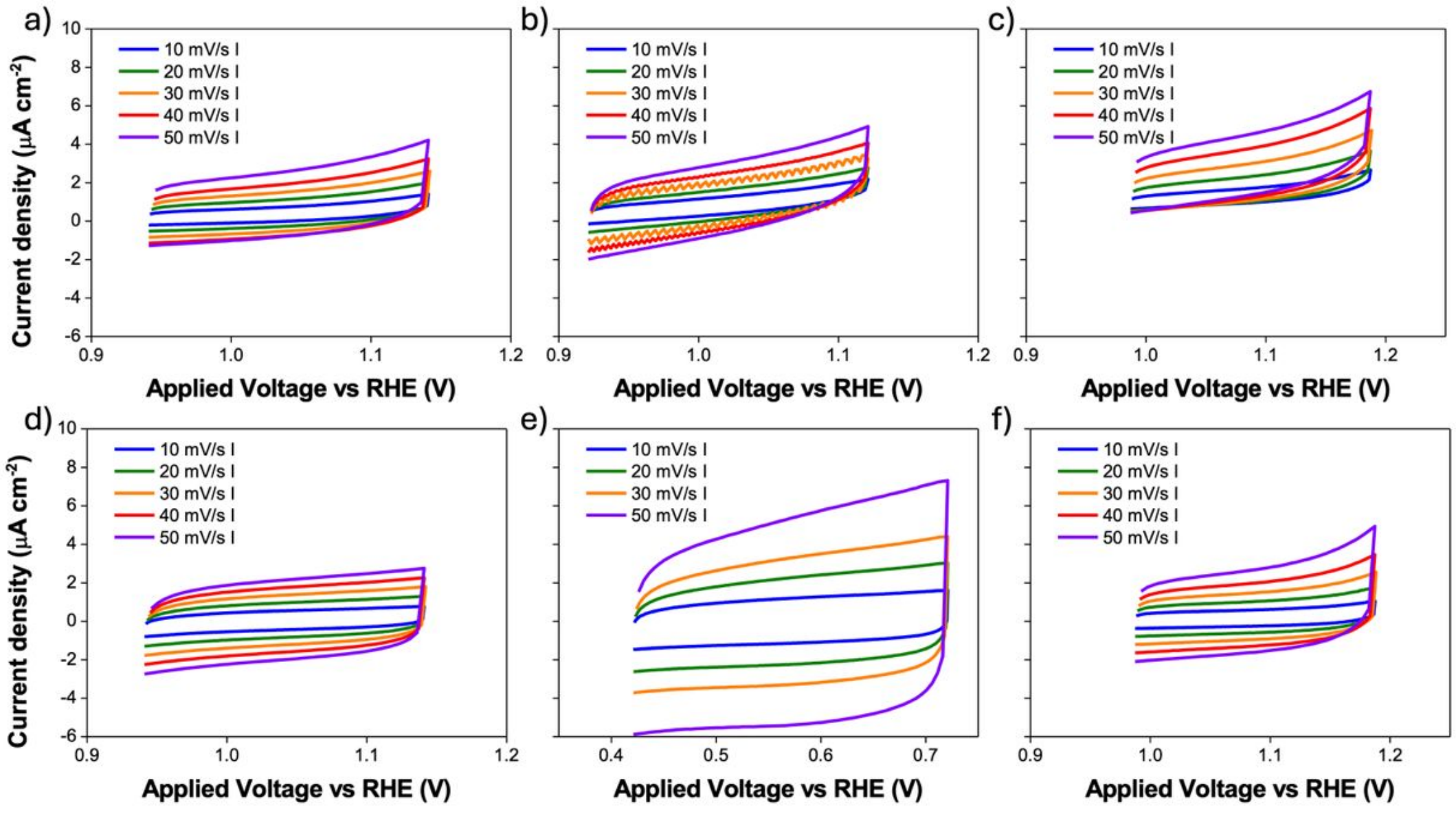

Figure S8. (a-c) CV curves at different scan rates for HC in alkaline, neutral, and acidic electrolytes, respectively; (d-f) CV curves for Pyro-HC under the same conditions.

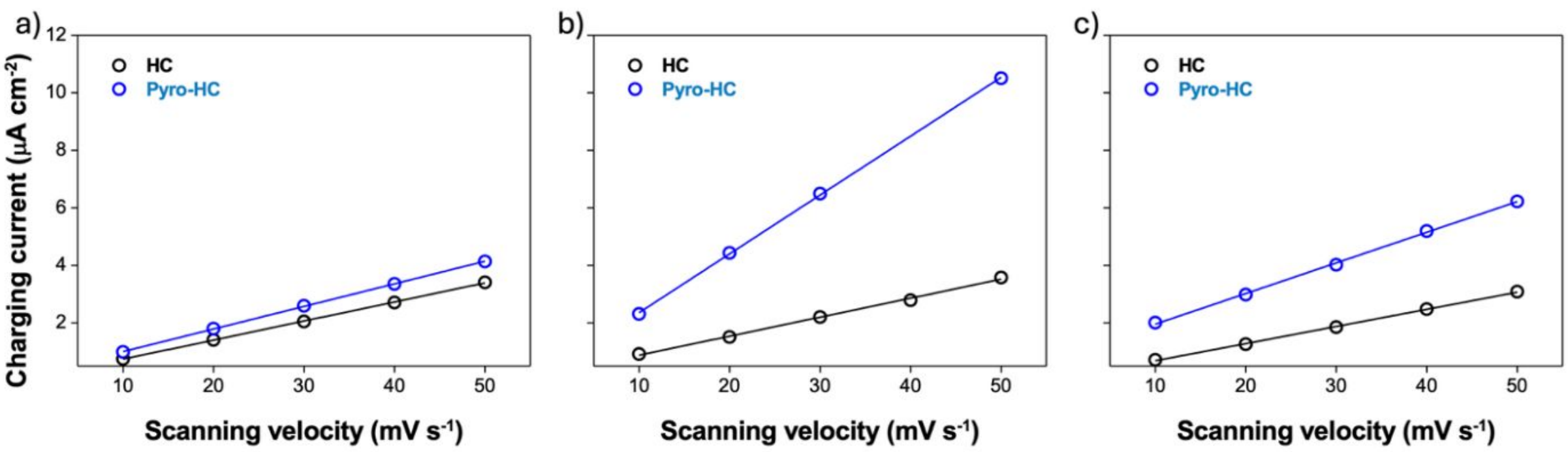

Figure S9. Charging current as a function of scan rate for HC and Pyro-HC in (a) alkaline, (b) neutral, and (c) acidic electrolytes.

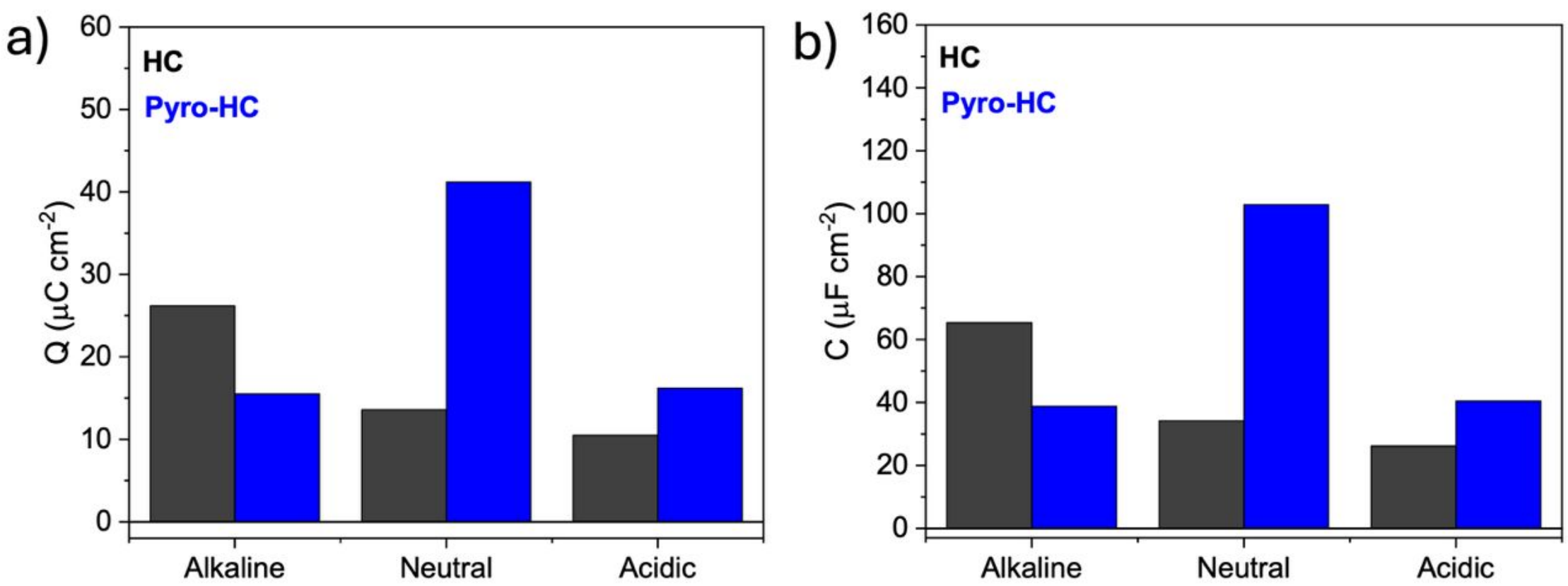

Figure S10. Comparison of non-Faradaic contributions: (a) charge and (b) capacitance for HC and Pyro-HC electrodes under different pH conditions.

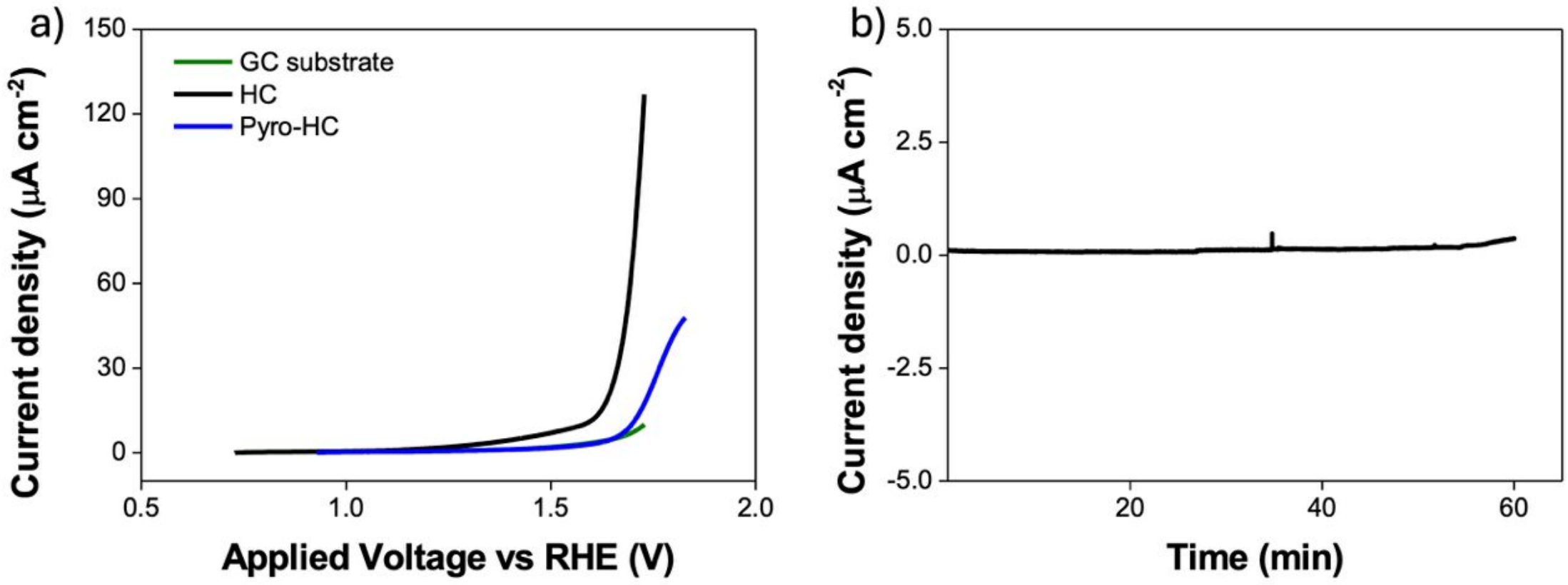

Figure S11. (a) LSV comparison of HC, Pyro-HC, and the glassy carbon substrate; (b) chronoamperometry at 0.95 V vs RHE to evaluate the long-term stability of HC in alkaline electrolyte.

Table S4. Electrochemical performance parameters of glucose-derived hydrochar and reference carbon materials.

Material	Tafel slope (mV dec^{-1})	Electrolyte	References
HC	108	0.1 M NaOH	Current work
Pyro-HC	129	0.1 M NaOH	Current work
rGO	80	0.1 M KNO_3	[6]

Table S5. Comparison of glucose- and saccharide-derived hydrochars reported in the literature with the material produced in this work.

Feedstock	HTC & post-treatment conditions	Particle size	Yield	Composition	Reported performance	Ref.
Glucose HC	HTC: 210 °C, 3 h, B/W = 0.5	12 ± 4 µm spherical morphology confirmed by SEM	SY = 46.9 ± 2.0%	H/C = 0.98; O/C = 0.35	67 µFcm^{-2} in 0.1 M NaOH	This work
Glucose Pyro-HC	HC produced at 210 °C, 3 h and B/W = 0.5; pyrolysis at 600 °C for 4 h under N_2	26 ± 14 µm spherical morphology confirmed by SEM	Post-treatment yield: N.R.	Bulk C, H and O: N.R.	40 µFcm^{-2} in 0.1 M NaOH	This work
Glucose-derived secondary hydrochar	HTC: 220 °C, 3 h; 1.11 M glucose; B/W = 0.25	0.25- 1 µm spherical morphology confirmed by STEM	SY = 48.4%	C = 67.4%; H = 4.5%; O = 26.7%; H/C = 0.81; O/C = 0.30	Electrochemical testing: N.R.	[7]
Glucose-derived hydrochar	HTC: 220 °C, pH 7, 1–12 h; 1.50 g glucose in 30 mL water	Stable-stage DLS size approximately 5.16–5.56 µm; spherical morphology confirmed by SEM	N.R.	Numerical bulk composition for the selected condition: N.R.	Electrochemical testing: N.R.	[8]
Microwave-derived glucose hydrochar	Microwave HTC: 200 °C, 45 min; 5 g glucose in 5 mL water	1–10 µm sphere-like microparticles of different sizes	SY = 40.03 ± 0.98%	C = 62.32 ± 1.80%; H = 4.31 ± 0.70%; O = 33.37 ± 1.10%; H/C = 0.82; O/C = 0.40	Electrochemical testing: N.R.; HHV = 21.30 MJ kg^{-1}	[9]
Glucose hydrochar, HC-G4	HTC: 210 °C, 4.5 h; glucose concentration = 0.50 M	1.2 ± 0.3 µm uniform micrometer-size spheres	SY = 28%	C = 66.29%; H = 4.15%; O = 29.56%; H/C = 0.752; O/C = 0.334	Electrochemical testing: N.R.	[10]
Sucrose-derived HC and pyrolyzed HC	HTC: 150 °C, 24 h; sucrose concentration = 0.50 M; pyrolysis at 400–1000 °C for 1 h under N_2	Broad microsphere distribution, from <1 to >10 µm; no representative global mean reported	HC yield = 32.1%; dry-HC mass loss during pyrolysis ≈42.9%	HC: C = 63.1%; H = 4.6%; O = 32.3%	Electrochemical testing: N.R.	[11]
Glucose-derived hydrothermal carbon	HTC: 220 °C, 4 h; glucose concentration = 0.10 M; no ammonium salt	Broad distribution of 1–10 µm; mean sizes for non-nitrogenated saccharide samples were 1.6–2.8 µm	SY = 21%	Bulk C, H and O: N.R. for G220	Electrochemical testing: N.R.	[12]
Glucose-derived carbon spheres	HTC: 200 °C, 4 h; 0.72, 1.44 or 7.28 g glucose in 14 mL water; heat treatment at 800 °C under Ar, with or without sulfonation	Numerical diameter: N.R.; interconnected and fused microsphere aggregates reported	Numerical HTC yield: N.R. in accessible text	C, H, O, H/C and O/C: N.R.	Best capacitance = 85 F g^{-1} at 50 mV s^{-1}; electrolyte: N.R. in accessible text	[13]
Urea-treated and KOH-activated glucose-	Glucose-derived hydrochar; urea thermal pretreatment	Numerical diameter: N.R.; spherical	HTC and activation yields: N.R.	C, H, O, H/C and O/C: N.R.; N = 12.9 at.% after treatment	354 F g^{-1} at 1 A g^{-1} and 264 F g^{-1} at 50 A	[14]

derived carbon spheres	followed by KOH activation; detailed parent HTC conditions: N.R. in accessible text	morphology retained			g^{-1} in 6 M KOH	
Glucose-derived micro–mesoporous carbon spheres	Glucose HTC followed by $ZnCl_2$ impregnation and high-temperature calcination; detailed temperatures and times: N.R. in accessible abstract	Numerical diameter: N.R.	HTC and post-treatment yields: N.R.	C, H, O, H/C and O/C: N.R.	Li–S battery: 1396 mAh g^{-1} initially at 0.1 C and 1014 mAh g^{-1} after 100 cycles; coulombic efficiency close to 100%	[15]
Oxidatively pretreated and KOH-activated glucose-derived carbon spheres	HTC: 200 °C, 4 h; glucose concentration ≈2.8 M; air calcination at 300 °C for 1 h; KOH impregnation at 100 °C; activation at 850 °C	Numerical diameter: N.R.; spherical morphology retained for the selected 6 M KOH-impregnated sample	Post-activation yield = 43.7%; this is not HTC SY	C, H, O, H/C and O/C: N.R.	263 F g^{-1} at 5 mV s^{-1} and approximately 220 F g^{-1} at 10 mV s^{-1}; electrolyte: N.R. in accessible text	[16]
ZIF-8-assisted glucose-derived carbon spheres	Optimal electrochemical sample: 3 M glucose, 40 mL solution, 0.1 g ZIF-8, 24 mg PAAS and 2 h HTC; KOH/carbon ratio = 1:1	Uniform carbon spheres; numerical diameter: N.R. in accessible text	Absolute SY: N.R.; apparent yield increased by nearly 10% relative to the control	C, H, O, H/C and O/C: N.R.	171.5 F g^{-1} at 0.5 A g^{-1}; 85.14% capacitance retention after 10,000 cycles at 2 A g^{-1}	[17]

N.R. = not reported in the cited study. The table is intended for contextual comparison only because reactor conditions, precursor concentration, separation procedures, and post-treatment protocols differ among the studies.

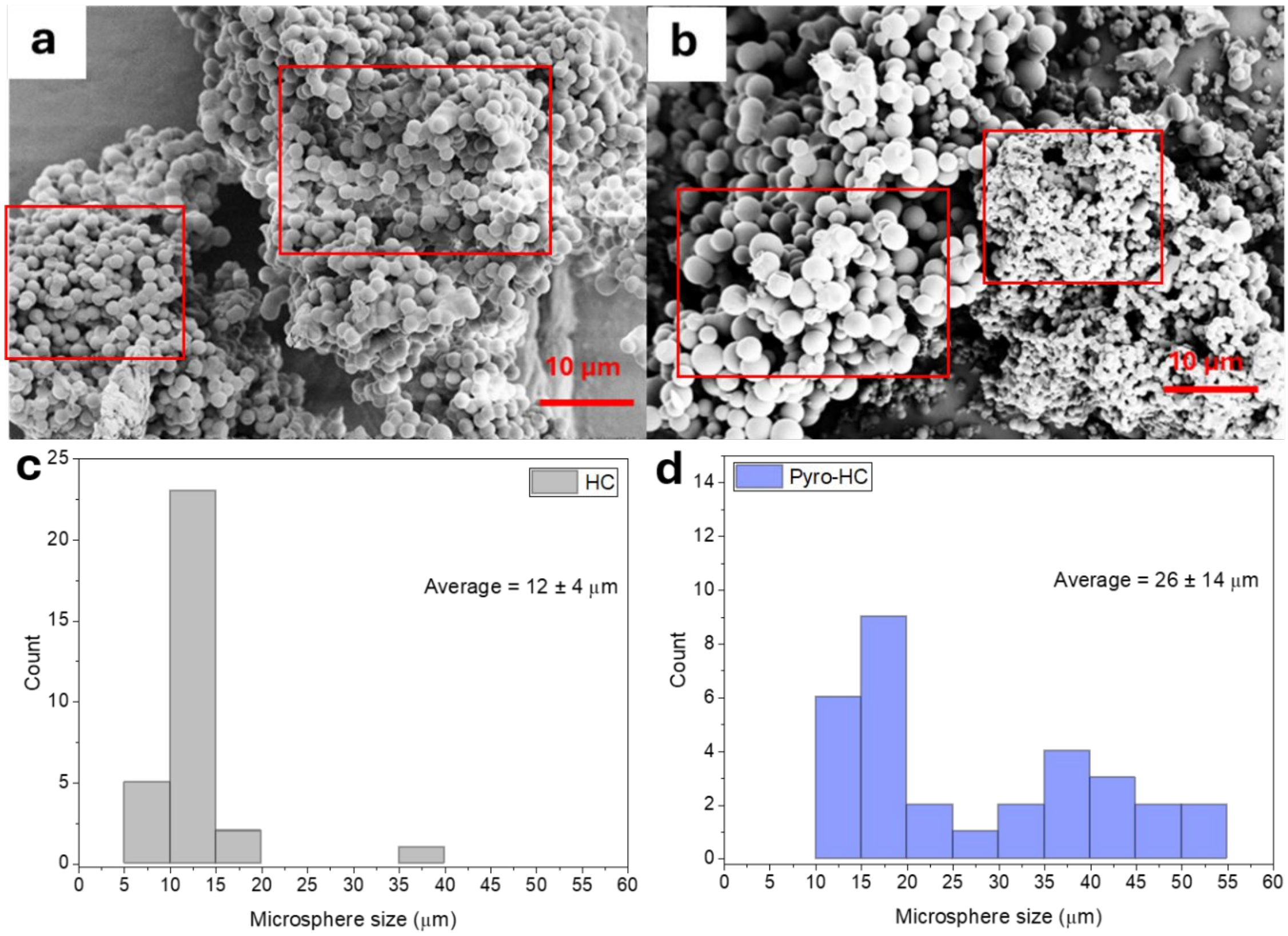

Figure S12. SEM images of hydrochar (a and b). Histograms of size distribution calculated based on analysis of SEM images and respective highlighted regions (c and d). Pyrolysis treatment at 600 °C preserves the microsphere characteristic of the hydrochar, with only minor variations in size distribution.

References

[1] M. Sevilla, A.B. Fuertes, The production of carbon materials by hydrothermal carbonization of cellulose, Carbon N. Y. 47 (2009) 2281–2289. https://doi.org/https://doi.org/10.1016/j.carbon.2009.04.026.

[2] M. Wortmann, W. Keil, B. Brockhagen, J. Biedinger, M. Westphal, C. Weinberger, E. Diestelhorst, W. Hachmann, Y. Zhao, M. Tiemann, G. Reiss, B. Hüsgen, C. Schmidt, K. Sattler, N. Frese, Pyrolysis of sucrose-derived hydrochar, J. Anal. Appl. Pyrolysis 161 (2022) 105404. https://doi.org/https://doi.org/10.1016/j.jaap.2021.105404.

[3] J. Peng, X. Kang, S. Zhao, P. Zhao, A.J. Ragauskas, C. Si, T. Xu, X. Song, Growth mechanism of glucose-based hydrochar under the effects of acid and temperature regulation, J. Colloid Interface Sci. 630 (2023) 654–665. https://doi.org/https://doi.org/10.1016/j.jcis.2022.10.044.

[4] N. Saha, M.T. Reza, Effect of pyrolysis on basic functional groups of hydrochars, Biomass Convers. Biorefin. 11 (2021) 1117–1124. https://doi.org/10.1007/s13399-019-00504-3.

[5] M. Sevilla, A.B. Fuertes, Chemical and Structural Properties of Carbonaceous Products Obtained by Hydrothermal Carbonization of Saccharides, Chemistry – A European Journal 15 (2009) 4195–4203. https://doi.org/https://doi.org/10.1002/chem.200802097.

[6] P.H.S. Borges, J. M. Gonçalves, C.B. Breslin, E. Nossol, Enhancing Oxygen Evolution Reaction Performance with rGO/CoNi-Prussian Blue-Derived Oxyhydroxide Nanocomposite Electrocatalyst: A Strategic Synthetic Approach, ACS Appl. Mater. Interfaces 16 (2024) 53705–53717. https://doi.org/10.1021/acsami.4c09452.

[7] G. Ischia, M. Cutillo, G. Guella, N. Bazzanella, M. Cazzanelli, M. Orlandi, A. Miotello, L. Fiori, Hydrothermal carbonization of glucose: Secondary char properties, reaction pathways, and kinetics, Chemical Engineering Journal 449 (2022). https://doi.org/10.1016/j.cej.2022.137827.

[8] J. Peng, X. Kang, S. Zhao, P. Zhao, A.J. Ragauskas, C. Si, T. Xu, X. Song, Growth mechanism of glucose-based hydrochar under the effects of acid and temperature regulation, J. Colloid Interface Sci. 630 (2023) 654–665. https://doi.org/10.1016/J.JCIS.2022.10.044.

[9] S.E. Elaigwu, G.M. Greenway, Chemical, structural and energy properties of hydrochars from microwave-assisted hydrothermal carbonization of glucose, International Journal of Industrial Chemistry 7 (2016) 449–456. https://doi.org/10.1007/S40090-016-0081-0/FIGURES/3.

[10] M. Sevilla, A.B. Fuertes, Chemical and Structural Properties of Carbonaceous Products Obtained by Hydrothermal Carbonization of Saccharides, Chemistry – A European Journal 15 (2009) 4195–4203. https://doi.org/10.1002/CHEM.200802097.

[11] M. Wortmann, W. Keil, B. Brockhagen, J. Biedinger, M. Westphal, C. Weinberger, E. Diestelhorst, W. Hachmann, Y. Zhao, M. Tiemann, G. Reiss, B. Hüsgen, C. Schmidt, K. Sattler, N. Frese, Pyrolysis of sucrose-derived hydrochar, J. Anal. Appl. Pyrolysis 161 (2022) 105404. https://doi.org/10.1016/J.JAAP.2021.105404.

[12] K.G. Latham, A. Ferguson, S.W. Donne, Influence of ammonium salts and temperature on the yield, morphology and chemical structure of hydrothermally carbonized saccharides, SN Appl. Sci. 1 (2019) 54-. https://doi.org/10.1007/S42452-018-0055-2/FIGURES/10.

[13] E. Tovar-Martinez, C.E. Sanchez-Rodriguez, J.D. Sanchez-Vasquez, M. Reyes-Reyes, R. López-Sandoval, Synthesis of carbon spheres from glucose using the hydrothermal carbonization method for the fabrication of EDLCs, Diam. Relat. Mater. 136 (2023). https://doi.org/10.1016/j.diamond.2023.110010.

[14] M.S. Wu, Y.T. Lin, J.P. Chen, Carbon spheres with ultra-hydrophilic surface and ordered pores on the borderline between micropores and mesopores for improving supercapacitive performance, J. Energy Storage 69 (2023) 107963. https://doi.org/10.1016/J.EST.2023.107963.

[15] C.Y. Xu, R. Du, C.B. Yu, Z.Y. Shi, J.L. Wang, T.R. Li, Glucose-Derived Micro–Mesoporous Carbon Spheres for High-Performance Lithium–Sulfur Batteries, Energy & Fuels 37 (2023) 1318–1326. https://doi.org/10.1021/ACS.ENERGYFUELS.2C03421.

[16] C.E. Sánchez-Rodríguez, E. Tovar-Martínez, R. López-Sandoval, Oxidative calcination-enhanced KOH activation of d-glucose-derived carbon spheres for high microporosity in supercapacitor electrodes, Electrochim. Acta 507 (2024) 145151. https://doi.org/10.1016/J.ELECTACTA.2024.145151.

[17] Y. Zhao, X. Tan, D. Wen, W. He, M. Cui, M. Wang, X. Liu, L. Hou, Zeolitic imidazolate framework-8-assisted synthesis of glucose-derived carbon spheres via hydrothermal carbonization for supercapacitors, Diam. Relat. Mater. 165 (2026) 113610. https://doi.org/10.1016/J.DIAMOND.2026.113610.
